# Gene Therapy for Acute Respiratory Distress Syndrome

**DOI:** 10.3389/fphys.2021.786255

**Published:** 2022-01-17

**Authors:** Jing Liu, David A. Dean

**Affiliations:** ^1^Department of Pediatrics, University of Rochester, Rochester, NY, United States; ^2^Department of Pharmacology and Physiology, University of Rochester, Rochester, NY, United States

**Keywords:** viral vectors, non-viral vectors, sepsis, acute lung injury, electroporation, alveolar fluid clearance, barrier function

## Abstract

Acute respiratory distress syndrome (ARDS) is a devastating clinical syndrome that leads to acute respiratory failure and accounts for over 70,000 deaths per year in the United States alone, even prior to the COVID-19 pandemic. While its molecular details have been teased apart and its pathophysiology largely established over the past 30 years, relatively few pharmacological advances in treatment have been made based on this knowledge. Indeed, mortality remains very close to what it was 30 years ago. As an alternative to traditional pharmacological approaches, gene therapy offers a highly controlled and targeted strategy to treat the disease at the molecular level. Although there is no single gene or combination of genes responsible for ARDS, there are a number of genes that can be targeted for upregulation or downregulation that could alleviate many of the symptoms and address the underlying mechanisms of this syndrome. This review will focus on the pathophysiology of ARDS and how gene therapy has been used for prevention and treatment. Strategies for gene delivery to the lung, such as barriers encountered during gene transfer, specific classes of genes that have been targeted, and the outcomes of these approaches on ARDS pathogenesis and resolution will be discussed.

## Introduction

Acute respiratory distress syndrome (ARDS) is a devastating clinical syndrome that leads to acute respiratory failure (Ware, 2006; Thompson et al., 2017; Matthay et al., 2019). ARDS can be directly caused by bacterial or viral infection of or chemical damage to the lung, or indirect due to injuries outside the lung or systemic inflammatory response, such as non-pulmonary sepsis, blood transfusions, and non-pulmonary injury (Pelosi et al., 2003; Rezoagli et al., 2017). In all cases, bacterial or viral infection is most commonly seen clinically. Most recently, the global pandemic of the coronavirus disease-2019 (COVID-19) has caused a high number of severe ARDS cases in the United States and around the world. As of October 24, 2021, there have been more than 45 million COVID 19 cases and more than 733,000 deaths in the United States (CDC, 2021). From multiple studies, approximately 33% of hospitalized COVID-19 patients develop ARDS, and there is a ∼70% mortality rate for COVID-19 patient-associated ARDS (Hasan et al., 2020; Tzotzos et al., 2020). The incidence of ARDS among non-survivors of COVID-19 is even higher, up to 90%, indicating that ARDS accounts for the majority of COVID-19 deaths (Tzotzos et al., 2020). Although the molecular mechanisms regarding the pathogenesis and progress of ARDS have been studied for decades, the development of effective treatments has lagged, and clinical management strategies still rely on supportive care, broad activity pharmacological treatment, ventilation, prone positioning, and other supportive strategies (Matthay et al., 2020). This review will focus on the pathophysiological features of ARDS and summarize the state of gene therapy treatments for ARDS.

## Cellular and Molecular Mechanisms of Acute Respiratory Distress Syndrome

**FIGURE 1:**
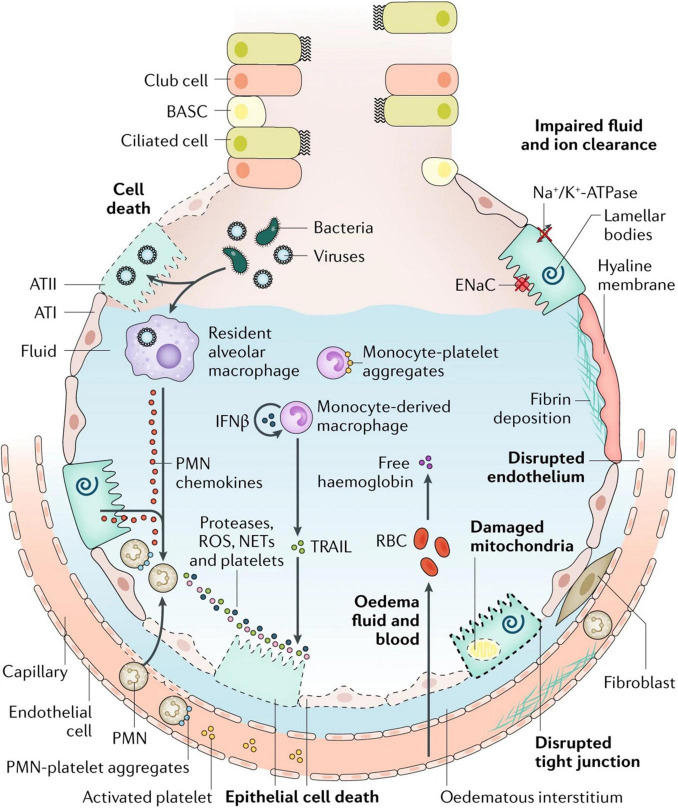
Injured alveolus in the acute phase of lung injury and acute respiratory distress syndrome. A variety of insults (such as acid, viruses, ventilator-associated lung injury, hyperoxia, and bacteria) can injure the epithelium, either directly or by inducing inflammation, which in turn injures the epithelium. Direct injury is inevitably exacerbated by a secondary wave of inflammatory injury. Activation of toll-like receptors (not shown) on alveolar type II (ATII) cells and resident macrophages induces the secretion of chemokines, which recruit circulating immune cells into the airspaces. As neutrophils migrate across the epithelium, they release toxic mediators, such as proteases, reactive oxygen species (ROS), and neutrophil extracellular traps (NETs), which have an important role in host defense but cause endothelial and epithelial injury. Monocytes also migrate into the lungs and can cause an injury, such as epithelial cell apoptosis *via* IFNβ-dependent release of tumor necrosis factor (TNF)-related apoptosis-inducing ligand (TRAIL), which activates death receptors. Activated platelets form aggregates with polymorphonuclear (PMN) leukocytes, which are involved in NET formation, and monocyte–platelet aggregates. Red blood cells (RBCs) release cell-free hemoglobin, which exacerbates injury *via* oxidant-dependent mechanisms. Angiopoietin 2 inhibits TIE2-stabilization of vascular endothelial cadherin (VE-cadherin); vascular endothelial growth factor and other permeability-promoting agonists also destabilize VE-cadherin *via* dissociation from p120-catenin, resulting in its internalization and enhanced paracellular permeability. Additionally, loss of cell–cell adhesion in the setting of actomyosin contraction results in the formation of occasional gaps between endothelial cells. Epithelial injury also includes wounding of the plasma membrane, which can be induced by bacterial pore-forming toxins or mechanical stretch, and mitochondrial dysfunction. Together, these effects result in endothelial and epithelial permeability, which further facilitates the transmigration of leukocytes and leads to the influx of edematous fluid and RBCs. Airspace filling with edematous fluid causes hypoxemia, resulting in the need for mechanical ventilation. Vascular injury and alveolar edema contribute to the decreased ability to excrete CO_2_ (hypercapnia), accounting for the elevated pulmonary dead space in acute respiratory distress syndrome. In turn, hypoxemia and hypercapnia impair vectorial sodium transport, reducing alveolar edema clearance. ATI, alveolar type I cell; BASC, bronchioalveolar stem cell; ENaC, epithelial sodium channel. Reproduced with permission (Ware and Matthay, 2000).

The key features of ARDS are pulmonary edema of noncardiogenic origin and pathologic diffuse alveolar damage (DAD; Thompson et al., 2017; Matthay et al., 2019), which is primarily caused by alveolar capillary barrier dysfunction and the resulting flooding of alveoli and lung interstitial space with protein-rich fluid (Matthay et al., 2019). The clinical hallmarks of ARDS include refractory hypoxemia due to insufficient gas exchange, fluffy bilateral infiltrates on X-ray radiographs, decreased lung compliance due to alveolar collapse and edema, and increased physiological dead space fraction due to lung microvascular destruction (Lewis and Jobe, 1993; Ware and Matthay, 2000; Nuckton et al., 2002; Matthay and Zemans, 2011). Although ARDS is defined by its pulmonary versus extrapulmonary origin, lung mechanical dysfunction is etiologically independent (Menezes et al., 2005). The observed edema and inflammation in both direct and indirect injured lungs indicate several significant events, namely, alveolar capillary barrier dysfunction, impaired alveolar fluid resolution, and uncontrolled neutrophil activation, sequestration, and their metabolite-mediated inflammatory responses (Ware and Matthay, 2001; Ware, 2006; Matthay and Zemans, 2011; Herrero et al., 2018). Perhaps the best illustration of these mechanisms remains the classic figure of the healthy and injured alveoli introduced by Lorraine Ware and Michael Matthay in 2000, although a relatively recent update incorporates several features now realized to be central to ARDS pathogenesis (Figure 1; Ware and Matthay, 2000). Understanding the mechanisms behind these events would provide insights to identify therapeutic targets for ARDS gene therapy.

### Alveolar-Capillary Barrier Dysfunction

One early event during the exudative phase that defines ARDS is the accumulation of pulmonary edema, the major factor causing hypoxemia (Box 1; Bhattacharya and Matthay, 2013; Matthay et al., 2019). In the normal lung, fluid homeostasis is maintained by microvascular filtration, which provides the fluid source, and lymphatic clearance, which drains the filtrate flow away (Bhattacharya and Matthay, 2013). Vascular endothelial permeability is the determining factor for microvascular filtration (Staub, 1978; Mehta and Malik, 2006). It shows selectivity to sieve large protein molecules (e.g., albumin) in the plasma (Siflinger-Birnboim et al., 1987; Mehta and Malik, 2006; Sukriti et al., 2014), creating high osmotic pressure to encounter the pro-filtration force (hydrostatic pressure) and promote water retention in the circulation (Bhattacharya and Matthay, 2013). Besides the lymphatic system, the filtrate also flows into alveoli forming a protective liquid layer with surfactant (Lindert et al., 2007). Alveolar fluid balance is mainly determined by epithelial integrity, which provides a tight barrier preventing fluid influx and alveolar fluid clearance (AFC), which drives water out of the alveolar space based on the Na^+^ osmotic gradient (Matthay et al., 2002; Johnson et al., 2006; Bhattacharya and Matthay, 2013; Herrero et al., 2018). The importance of the alveolar-capillary barrier and AFC can be reflected from the cardiogenic lung edema, which shows much less protein content (Sprung et al., 1981; Ware et al., 2010), and could be quickly resolved because of the relatively intact alveolar epithelial and capillary endothelial integrity and fluid clearance capacity (Matthay, 2014; Hamacher et al., 2018). However, in ARDS, accumulated pulmonary edema results from the loss of the alveolar-capillary barrier, hyperpermeability, and impaired AFC, allowing the influx of fluid and large amounts of proteins to accumulate in the interstitial and alveolar spaces (Ware and Matthay, 2000, 2001; Matthay et al., 2012; Matthay, 2014). In patients with ARDS, various endothelial injury markers, e.g., von Willebrand factor (VWF; Ware et al., 2004), can be detected in blood and epithelial apoptotic markers, e.g., cytokeratin-18 (Lee et al., 2008; Galani et al., 2010), and can be measured in bronchoalveolar lavage (BAL) fluid. Clinically, the decreased AFC capacity is associated with prolonged acute respiratory failure and higher mortality rate (Ware and Matthay, 2001); and the degree of injury to the alveolar epithelium appears to be a determinant of the severity of ARDS (Matthay et al., 2005; Yanagi et al., 2015). Multiple mechanisms are involved in alveolar-capillary barrier and AFC dysfunction, such as cell death, loss of cell-cell adhesion molecules and ion transporter activities, and activation of neutrophils and their products. Apoptosis induces alveolar epithelial cell death, and many studies show that apoptosis is the main route of cell death detectable immediately following lung injury (Albertine et al., 2002; Martin et al., 2003; Tang et al., 2008; Perl et al., 2010; Herrero et al., 2013; Jagrosse et al., 2019). In addition, cellular necrosis is also seen in patients with ARDS (Tomashefski, 2000; Cardinal-Fernández et al., 2017). Many studies have shown that Fas/Fas ligand (FasL) extrinsic pathway-mediated apoptosis contributes to ARDS (Herrero et al., 2013). The Fas/FasL system is significantly upregulated in the pulmonary edema fluid of patients with ARDS and is associated with increased mortality (Matute-Bello et al., 1999; Albertine et al., 2002). In animal models of ARDS, there is increased expression of Fas in epithelial cells and FasL in BAL (Fine et al., 1997; Hamann et al., 1998; Perl et al., 2007). A study using chimeric mice expressing Fas receptor exclusively on non-myeloid cells, including lung epithelial cells, demonstrated that lung injury is primarily via the activation of the pro-apoptotic pathway in alveolar epithelial cellsand is associated with increased alveolar permeability and edema formation (Matute-Bello et al., 2005a). Besides the Fas/FasL-mediated extrinsic apoptotic pathway, Bcl-2-mediated intrinsic apoptosis is also involved, which might be relevant to the mitochondrial dysfunction seen in this disease (Tang et al., 2008). During ARDS, cell death is greatly due to multiple cellular dysfunctions: elevated CO_2_ levels and oxidative imbalance lead to mitochondrial DNA damage and cell toxicity (Herold et al., 2013), ventilator-induced mechanical stress (Syrkina et al., 2008), hypoxia (Tang et al., 2008), NO formation by iNOS (Rudkowski et al., 2004), and LPS-activated apoptotic signaling (Zeng et al., 2018). Since gene therapy requires living cells as targets in order for any gene expression to take place, the level of apoptotic or necrotic cells can have a profound effect on any such treatment. Thus, in order for gene therapy to be effective, the level of cell death needs to be relatively low or the treatment must be administered prior to significant cell loss.

**Box 1. Mechanisms for edema formation.**

I) **Alveolar-capillary barrier dysfunction**
  • **Epithelial and endothelial cell injury markers:**
    Fas/FasL, RAGE (epithelial)
    VWF, Angiopoietin 2 (endothelial)
  • **Cell death:** apoptosis; necrosis
  • **Adhesion junction disruption and downregulation:** Proteolytic degradation: ZO-1; VE-cadherin; E-cadherin Claudin-4,5,18 Phosphorylation and internalization: occludin; VE-cadherin
  • **Inducing factors:** oxidative stress; ROS; ventilation; mitochondrial damage NETs; cytokines; LPS; virus

II) **Alveolar fluid clearance dysfunction**
  • **Decreased cell membrane abundance of Na, K, ENaC** hypoxia; ROS; IAV; IFNs; TRAIL; coagulation proteases; decreased adrenergic stimulation
  • **Decreased mRNA and protein level of Na, k, ENaC, CFTR** hypoxia; IL-lb; TGFbl; TNFa; oxidant; INFr
  • **Decreased transepithelial ion transport activities** loss of epithelial polarity reduced channel open probability

III) **PMN activation and inflammation mediators**
  • **Increased PMN recruitment**
  • **NETosis induced lung barrier disruption** DNA; histone; MPO; NE
  • **extracellular matrix modeling** increased MM P-2,9
  • **Increased cytokine level in plasma and BAL fluid** IL-lb, TNFa, IL-6 and IL-8

The alveolar capillary barrier is composed of two physical barriers: a tight alveolar epithelial monolayer of flat ATI cells (95% of alveolar surface area) and cuboidal ATII cells (5% of alveolar surface area), and a relatively more permeable microvascular endothelial cell monolayer (Bhattacharya and Matthay, 2013; Knudsen and Ochs, 2018; Katz et al., 2019). A study measuring (Matthay and Zimmerman, 2005) I-labeled albumin flux in blood, surrounding interstitium, and alveolar space in sheep lungs indicates that more than 92% of resistance to albumin flux across the alveolar capillary barrier lies in the epithelial barrier (Gorin and Stewart, 1979). Adhesion molecules holding together neighboring cells in the monolayer are the major structural components regulating the paracellular permeability pathway, the major route for passage of large molecules, such as albumin, across both barriers (Mehta and Malik, 2006; Overgaard et al., 2012; Bhattacharya and Matthay, 2013). Tight junctions (TJs) are located in the most apical side of the alveolar epithelium and largely determine barrier tightness (Overgaard et al., 2012; Gunzel and Yu, 2013). Damage to TJs greatly contributes to epithelial barrier leakage, further increasing edema accumulation without necessary cell death (Hook et al., 2018; Matthay et al., 2019). Claudins, key tight junction proteins, are highly expressed in the alveolar epithelium with the predominant isoforms being claudin-3, 4, and 18 (Overgaard et al., 2012). Knockout (KO) of claudin18 in mice shows significant accumulation of FITC-albumin in the BAL 4 h after intraperitoneal instillation of labeled tracer, indicating increased alveolar epithelial permeability (LaFemina et al., 2014). Morphological disruption of this barrier is further confirmed by ultrastructural analysis of ATI and ATII cells (Bachofen and Weibel, 1977, 1982). In cultured alveolar epithelial cells, silencing of claudin 18 by siRNA shows decreased transepithelial electrical resistance (TEER), a measurement of epithelial tightness *in vitro*, and increased permeability to small size tracer markers (LaFemina et al., 2014; Srinivasan et al., 2015). Similarly, overexpression of claudin 4 increases TEER by nearly 50% (Mitchell et al., 2011); KO of claudin 4 in mice not only increases barrier permeability to solute but also, surprisingly, decreases AFC, which might be relevant to decrease in Na^+^, K^+^-ATPase activity (Wray et al., 2009; Overgaard et al., 2012). The promotion of AFC by claudin 4 in the alveolar epithelium is also indicated by the property of Cl^–^-selective paracellular permeability, since claudin4 limits paracellular Na flux but favors transepithelial Cl^–^ transport for electrical neutrality during Na^+^ active transport for fluid clearance (Colegio et al., 2002, 2003). Occludin is another important transmembrane a TJ molecule expresses in both the epithelium and endothelium (Förster, 2008). The internalization and phosphorylation of occludin are associated with lung barrier dysfunction (Hirase et al., 2001; Förster, 2008). TJ proteins are targets of numerous factors during ARDS, namely, excessive ROS (Rao, 2008), pathogens (Lu et al., 2014), e.g., LPS, viruses and bacteria, ventilation (Liu et al., 2014), inflammatory mediators (Al-Sadi et al., 2009), hypoxia (Caraballo et al., 2011), hyperoxia (You et al., 2012), and inhaled particulate matter (PM; Wang et al., 2012).

Endothelial dysfunction is another important contributor to alveolar capillary barrier disruption, leading to uncontrolled extravascular fluid leakage. The microvascular endothelium is the first barrier encountered by fluid and neutrophils infiltrating from vessels into the alveoli. Under normal conditions, the endothelium is more permeable to large macromolecules than the alveolar epithelium. However, upon activation by pathogens, e.g., LPS-containing bacteria, the endothelial barrier properties are altered by a series of events, such as structural damage to the endothelial barrier, significant proinflammatory response, coagulation and micro-thrombosis formation, and vascular tone dysregulation (Vassiliou et al., 2020).

Endothelial hyperpermeability can directly result from structural damage to the endothelial barrier, through both endothelial cell apoptosis and inter-endothelial cell junctional complex disruption. For the former, mitochondrial DNA (mtDNA) damage may initiate endothelial cell death (Ruchko et al., 2005). Mitochondria are a major source of ROS in the endothelium (Ince et al., 2016). Under oxidative stress, mtDNA released from mitochondria, in turn, triggers mitochondrial dysfunction and induces apoptosis through cytochrome c and the intrinsic apoptosis pathway. Circulating mtDNA has been reported as a plasma biomarker for the severity of sepsis or sepsis-related ARDS with higher plasma cell-free mtDNA levels observed in ICU patients who died within 28 days of medical ICU admission, as well as in ICU patients with sepsis or ARDS (Nakahira et al., 2014). Receptor agonism-initiated extrinsic apoptosis also contributes to endothelial cell death. Tumor necrosis factor alpha (TNFα) receptor and Fas are also expressed in endothelial cells, and their activation has been shown to induce caspases 8 and 3 signaling, resulting in apoptosis (Hotchkiss et al., 2002). Recently, more types of cell death programs have been identified in dysfunctional endothelium during sepsis, such as necrosis and pyroptosis (Singla and Machado, 2018). Pyroptosis is of particular interest in sepsis, since it is triggered by proinflammatory signals and is vital for endothelial injury when overactivated (Gao et al., 2018). Knockout of one of key pyroptosis mediators, such as caspase 1 or 11, displays resistance to endotoxic shock in mice and provides increased survival and protection against vascular injury, endothelial hyperpermeability, lung edema, and histological damage (Li et al., 1995; Cheng et al., 2017; Mitra et al., 2018).

The endothelial paracellular pathway is the major filtration route of the microvasculature, and disruption of inter-endothelial junction structures may account for another mechanism of barrier hyperpermeability (Bhattacharya and Matthay, 2013). Inter-endothelial junction molecules include tight junctions, adherens junctions, junctional adhesion molecules (JAMs), and other endothelial specific molecules, such as platelet endothelial cell adhesion molecules (PECAMs; Komarova and Malik, 2010). In contrast to the alveolar epithelium where tight junctions play a major role in the integrity of epithelial barrier function, in the capillary endothelium, tight junctions are secondary, while adherens junctions play a more significant role (Mehta and Malik, 2006; Aird, 2007). Vascular endothelial cadherin (VE-cadherin), a key component of the endothelial adherens junction, primarily maintains the architectural integrity of the endothelial barrier, rendering high permeability to plasma proteins, a key property in establishing protein (e.g., albumin) gradients for fluid balance in the lungs. Claudin-5 is the predominant tight junction molecule expressed in pulmonary endothelial cells (Kaarteenaho-Wiik and Soini, 2009) and has been found to be downregulated in various models of ALI, such as influenza infection (Armstrong et al., 2012). Pathogen-induced VE-cadherin phosphorylation, internalization, and lysosomal degradation are major forms of VE-cadherin disruption (Chan et al., 2020). All of these junctional molecules are also targets of oxidative stress, ROS, IL-1β and other stimuli (Xiong et al., 2020). In addition, some sepsis mediators, e.g., high-mobility group protein B1, have been shown to activate acto-myosin contraction, inducing endothelial cell retraction, which mechanically breaks apart adhesion junctions, leading to hyperpermeability (Wolfson et al., 2011). Such cytoskeletal contraction due to phosphorylation of myosin light chain (MLC) is a common cause of endothelial cell retraction, and signaling pathways involved in the activation of MLC have been extensively studied and shown to be mediated through a number of pathways, most notably RhoA/ROCK (Reutershan et al., 2007). Furthermore, influx of Ca2+ has been shown to increase vascular permeability and allow for migration of neutrophils across the alveolocapillary barrier, as well as overall vascular leakage (Alvarez et al., 2006).

Glycocalyx shedding has been recognized in recent years as another crucial mechanism undermining endothelial barrier integrity, leading to edema formation and sepsis-induced organ failure. The glycocalyx is a thin multicomponent fibrous matrix layer lining the luminal endothelial surface, which includes proteoglycans, glycoproteins, and glycosaminoglycans that protect the vascular endothelium from oxidants, hyperglycemia, cytokines, and bacterial endotoxins (Weinbaum et al., 2007; Ince et al., 2016). However, these toxins, in turn, often induce glycocalyx degradation, and that layer becomes thinner as a result of and during sepsis. Glycocalyx fragments, such as syndecan-1, shed into the blood and have been reported as potential clinical biomarkers for sepsis survival and respiratory failure (Smart et al., 2018). Bacteria, TNF-α, and ROS all induce degradation of the glycocalyx layer, making the endothelial lining more vulnerable to pathogens and leading to barrier disruption and protein-rich extravascular fluid leakage. That the glycocalyx provides such a protective layer to the endothelium is exemplified by the fact that Crocin, a chemical compound, has been shown to prevent LPS-induced ARDS by protecting against glycocalyx degradation (Zhang et al., 2020). Glycocalyx shedding also decreases the sensitivity of endothelial cell responses to sheer stress, leading to unbalanced release of nitric oxide and vascular tone dysregulation (Ince et al., 2016). In addition, glycocalyx shedding may exacerbate endothelial proinflammatory response by promoting neutrophil adhesion to endothelial cells.

Neutrophil activation and transmigration from the circulation into lung tissue are perhaps the most significant events of the proinflammatory response during the early stage of ALI. However, excessive neutrophil activation induces endothelial barrier damage and, ultimately, lung damage. Neutrophil transendothelial migration requires temporal and spatial increases in endothelial paracellular permeability, which is a process found in normal host defense. However, uncontrolled neutrophil transmigration results in the prolonged opening of intercellular junction structures and increased paracellular permeability, which leads to fluid accumulation and edema in the interstitial tissue, and, ultimately, ARDS (Tsushima et al., 2009). Moreover, toxic mediators (e.g., proteases and ROS, etc.) and cytokines released from activated neutrophils also damage endothelial cells, inducing vascular leakage. More details are addressed below in “Neutrophil activation and inflammatory mediators.” In addition, endothelial homeostasis is disrupted during sepsis or ARDS, shifting to a pro-coagulant condition with massive production of thrombin, which directly affects the endothelial barrier, leading to hyperpermeability (Bogatcheva et al., 2002).

### Alveolar Fluid Clearance Dysfunction

Alveolar fluid clearance, or AFC, is important for maintaining fluid homeostasis in the lungs and is regulated by osmotic pressure (Matthay et al., 2002; Huppert and Matthay, 2017). The active transport of Na^+^ ions is the main contributor for the creation of the osmotic gradient: Na^+^ is primarily transported through the amiloride-sensitive ENaC, as well as by the nonselective (NCC) or highly selective cation channels (SCC) and cyclic nucleotide-gated (CNG) channels on the apical epithelial surface and then extruded out of the cell by the Na^+^, K^+^ ATPase on the basolateral surface into the interstitium and the circulation (Matthay, 2014). The Na^+^, K^+^ ATPase is the primary determining factor for AFC since it is the major active Na^+^ transporter expressed in the epithelial basolateral membrane, which continuously pumps Na^+^ out of the alveoli by utilizing ATP (Mutlu and Sznajder, 2005). Instillation of ouabain, a cardiac aminoglycoside inhibitor of the Na^+^, K^+^ ATPase, into animal and *ex vivo* human lungs decreases AFC by more than 50% (Matthay et al., 2002). Conversely, adenovirus- or electroporation-mediated overexpression of the Na^+^, K^+^ ATPase increased AFC by ∼100% in rat lungs (Machado-Aranda et al., 2005).

During lung injury, fluid clearance is impaired, ultimately resulting in hypoxemia (Huppert and Matthay, 2017). Majority of patients with ARDS show severe fluid clearance impairment, whereas 25% of all patients with hydrostatic lung edema have ARDS (Ware and Matthay, 2001). Multiple factors result in AFC impairment during ARDS (Vadasz et al., 2007). Loss of epithelial polarity due to disrupted epithelial TJs decreases AFC (Han et al., 2004; Zemans and Matthay, 2004). Hypoxia induces the downregulation of both ENaC and Na^+^, K^+^ ATPase at the mRNA level and membrane abundance of the Na^+^, K^+^ ATPase (Planès et al., 1997; Zhou G. et al., 2008). The Na^+^, K^+^ ATPase is most vulnerable to hypoxic effects, since it works by consuming ATP. Indeed, ∼40% of a cell’s total energy is consumed by this transporter to maintain homeostasis, and during injury, reduced oxygenation limits ATP production (Milligan and McBride, 1985). Excessive resulting ROS triggers Na^+^, K^+^ ATPase endocytosis through α1 subunit phosphorylation (Dada et al., 2003). Pathogens, such as influenza A virus, induce degradation of membrane-localized Na^+^, K^+^ ATPase in nearby noninfected alveolar epithelial cells by activating pathways in the infected epithelium and resident macrophages that produce cytokines like type I IFN and IFN- related apoptosis-inducing ligand (TRAIL; Peteranderl et al., 2016). Proinflammatory cytokines, such as IL-1β, IL-8, and TGFβ1, are detected at high levels in the edema fluid of patients in the early stage of ARDS (Pugin et al., 1999; Lee et al., 2011). They downregulate the expression and function of ENaC and the Na^+^, K^+^ ATPase through activation of various pathways in the epithelium, causing decreased AFC (Pugin et al., 1999). Furthermore, mechanical ventilation also negatively affects Na^+^ transport activity and AFC (Lecuona et al., 1999), although it is commonly used in the ICU to facilitate breathing and oxygenation. Indeed, in ATII cells isolated from rats after high tidal volume ventilation, the activity of Na^+^, K^+^ ATPase decreased by 50% compared to the control group (Lecuona et al., 1999).

**FIGURE 2:**
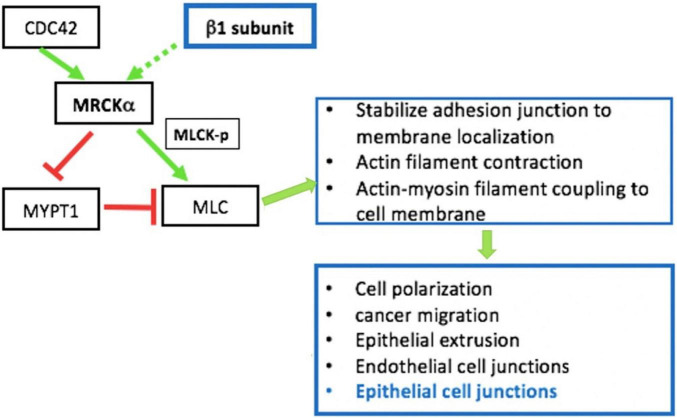
MRCKα signaling pathway is involved in epithelial intercellular junction regulation. MRCKα is activated (solid green arrow) by cdc42, a Rho family of small GTPases. The MRCKα kinase activates myosin light chain (MLC) either by directly phosphorylating/activating (green arrow) myosin light chain kinase (MLCK) or by inactivating (red arrow) myosin phosphatase target subunit 1 (MYPT1), which dephosphorylates/inactivates (red arrow) MLC. The two consecutive negative activities (red arrows) result in the indirect activation of MLC by reducing MLC dephosphorylation/inactivation. These events regulate multiple cellular functions such as actin-myosin contraction, which is involved in epithelial junction regulation. In addition, our previous data indicate that overexpression of β1- Na^+^, K^+^-ATPase increases activation/phosphorylation of MLC (Bai et al., 2021).

Besides being critical for AFC, the expression and function of the Na^+^, K^+^ ATPase are closely involved in the regulation of epithelial barrier integrity (Rajasekaran et al., 2001b; Rajasekaran and Rajasekaran, 2003; Vadasz et al., 2007). Increasing evidence indicates that the role of the Na^+^, K^+^ ATPase in barrier junction formation is independent of its ion transport activity. For example, (1) the Na^+^, K^+^ ATPaseβ1 subunit may be necessary for membrane localization of TJs (Rajasekaran et al., 2007); silencing of β1 by siRNA disrupts the continuous staining pattern of ZO-1 and occludin, indicating that β1 might be directly or indirectly associated with TJs (Madan et al., 2007); (2) the Na^+^, K^+^ ATPase is required for establishing epithelial polarization in MDCK cells and co-expression of the Na^+^, K^+^ ATPaseβ1 subunit, and E-cadherin recovers the lost polarity and junctions seen in MSV-MDCK cells, a highly invasive cell line (Rajasekaran et al., 2001b); (3) the basolateral localized Na^+^, K^+^ ATPase also acts as an adhesion molecule, forming a trans-dimer junction structure mediated through the N-glycosylation of β1’s extracellular domain (Vagin et al., 2012). The mechanisms by which the Na^+^, K^+^ ATPase regulates epithelial barrier function has not completely been characterized, but it appears related to stress fiber formation and actin assembly (Rajasekaran and Rajasekaran, 2003). RhoA GTPase, a small GTP-binding protein involved in stress fiber formation (Guasch et al., 1998), has been implicated as a downstream effector of Na^+^, K^+^ ATPase signaling for TJ assembly and function (Rajasekaran et al., 2001a). We recently identified MRCKα (CDC42-binding protein kinase alpha) by mass spectrometry as an interacting partner of the β1 subunit (Bai et al., 2021). These findings point to the interdependency of alveolar capillary barrier and AFC dysfunction in edema formation during ARDS. MRCKα is a Rho GTPase effector kinase that regulates diverse cell behaviors, such as actomyosin contraction-mediated junction formation (Figure 2; Unbekandt and Olson, 2014). Silencing of MRCKα by siRNA abolished the increased TEER seen in cultured AT1 cells following transfection with the β1 subunit, indicating that β1 signals through MRCKα to upregulate tight junction proteins and epithelial barrier function, at least in cells (Bai et al., 2021).

### Neutrophil Activation, Inflammatory Mediators, and Coagulation

Recruitment of neutrophils is a hallmark of ARDS and is considered to play a key role in the progression of ARDS. An analysis of BAL fluid cellularity in patients with ARDS or animals in various injury models demonstrates elevated neutrophil infiltration into alveoli. This is correlated with ARDS outcomes and severity, with non-survivors having higher levels of chemotactic IL-8 than survivors of ARDS. Neutrophils are important components of the innate immune system. In response to stimuli, they are activated, recruited, and secrete various antimicrobial molecules, such as ROS, proteases, and cationic peptides, to destroy invading microorganisms. However, under some disease conditions, e.g., ARDS, excessive neutrophil activation and imbalanced inflammatory responses can cause additional tissue damage. NETosis is an important mechanism of neutrophil defense against invading pathogens, in which neutrophils release DNA combined with histones, myeloperoxidase, neutrophil elastase, and extracellular fibers into the extracellular environment to form a network for microorganism trapping (Thiam et al., 2020). This increased extracellular NET production is correlated with ARDS severity and alveolar capillary barrier dysfunction in experimental models. Elastase in NETs degrades alveolar capillary barrier integrity, contributing to edema formation. It proteolytically degrades junctional molecules, such as ZO-1, E-cadherin, and VE-cadherin, to induce intercellular adhesion disruption in cultured epithelial and endothelial monolayers. Histones are the most abundant proteins in NETs, and it has been shown that incubation of endothelial cells or ATII cells with histones induces cell death, suggesting a role in damage to alveolar capillary barrier integrity. Finally, matrix metalloproteinases (MMPs) are produced by a variety of cell types, such as neutrophils, and the levels of MMP-2 and 9 are increased in BAL of patients and are correlated with ARDS severity.

Uncontrolled inflammatory response is another hallmark of the early stage of ARDS that contributes to lung barrier disruption and AFC impairment, leading to edema formation (Ware and Matthay, 2000; Matthay and Zimmerman, 2005). Inflammatory response is initiated, amplified, and regulated by a network of various cytokines and other inflammatory molecules (Park et al., 2001). High concentrations of cytokines, such as IL-1β, TNFα, IL-6, and IL-8, are detected in plasma and BAL fluids and are associated with poor clinical outcomes of ARDS, such as mortality rate (Pugin et al., 1996; Parsons et al., 2005; McClintock et al., 2008). In several experimental models, highly expressed TNFα in BAL fluid triggers caspase-8-mediated apoptotic signaling in the epithelium, inducing cell death, and consequently, alveolar epithelial barrier dysfunction (Patel B. V. et al., 2013).

Coagulation is a critical host response to infection. However, it is also involved in ARDS pathogenesis with markedly increased release of soluble tissue factor and microthrombi formation in the pulmonary microvasculature and decreased fibrinolytic activities with diffuse alveolar and interstitial fibrin deposition (Grinnell et al., 1980; Nagy et al., 1995). Some studies have shown that pro-coagulant activities observed during ARDS can increase alveolar capillary barrier permeability (Abraham, 2000; Mosnier et al., 2007). As seen in both patients and experimental animal models, upregulated levels of soluble tissue factor, thrombin, and fibrinolysis inhibitors are present in BAL fluid (Idell et al., 1991; Fuchs-Buder et al., 1996). In cultured epithelial cells, thrombin induces F-actin polymerization and stress fiber formation, which further increases cell contraction, stiffness, and epithelial barrier permeability (Hayashi et al., 2006). Platelets are another important component of the host defense system and may contribute to the neutrophil-dependent lung injury (Rossaint et al., 2018). Activated platelets have been shown to directly interact with neutrophils, facilitating their extravasation and recruitment to the lungs (Zarbock and Ley, 2009; Rossaint et al., 2018). In acid-induced lung injury, platelet-neutrophil interactions in the vascular endothelium could be visualized by electron microscopy within 30 min of injury (Zarbock et al., 2006). These studies showed that platelet depletion significantly reduces neutrophil rolling and adherence to the endothelium, thereby markedly reducing lung edema and increasing mouse survival.

### Current Pharmacological Treatments for Acute Respiratory Distress Syndrome

Over the past several decades, considerable research efforts have made ARDS well-understood in terms of pathogenesis, risk factors, genetic predispositions, and various signaling pathways and molecules involved. Although large efforts have been committed to developing pharmacological therapies for ARDS, the results have been discouraging. A review from 2018 summarized that large-scale clinical trials with positive results only account for 5% of the 20 most recent large pharmacological studies on both sepsis and ARDS (Laffey and Kavanagh, 2018). The incidence and overall hospital mortality of ARDS have not changed considerably in the past 10 years (Villar et al., 2016). In the 2016 cross-country study LUNG SAFE, the mortality rate remained at 40% for moderate ARDS and even higher at 46% for severe ARDS (Bellani et al., 2016). In the evolving COVID-19 pandemic, the mortality rate for patients with COVID-19 associated ARDS is even higher, which further highlights the importance of developing novel treatments or therapies for ARDS.

β_2_ Adrenergic agonists have been demonstrated to enhance AFC *in vivo* through the activation of the cAMP pathway, which increases transepithelial ion transport by upregulating the activity and membrane abundance of ENaC, Na^+^, K^+^-ATPases and chloride channels (Bertorello et al., 1999; Matalon and O’Brodovich, 1999; Fang et al., 2002; Mutlu et al., 2004; Mutlu and Sznajder, 2005). While treatment of mice with existing acute lung injury with several different β_2_ agonists, such as albuterol and salmeterol, can treat the disease and give positive outcomes in lung function, inflammation, edema clearance, and survival, none of these treatments have been proven effective in patients. Indeed, it has even been suggested to avoid using these drugs in patients with ARDS (Boyle et al., 2013). In several clinical studies, β_2_ adrenergic therapy showed no significance in the primary outcome of ventilator-free days and even worsened the outcome of increased mortality, although there was some amelioration in pulmonary fluid accumulation (Perkins et al., 2006; National Heart Lung Blood Institute Acute Respiratory Distress Syndrome (ARDS) Clinical Trials Network et al., 2011; Gao Smith et al., 2012; Matthay et al., 2017; Laffey and Kavanagh, 2018). Several possibilities might account for the failure of β_2_ receptor agonist therapy. For example, prolonged β_2_ adrenergic agonism by endogenous catecholamines could desensitize β_2_ receptors, which would prevent further receptor stimulation with exogenous catecholamines (Berthiaume et al., 2002). In some patients with no response to β adrenergic therapy, the alveolar epithelium might be too injured to benefit from any transporter upregulation (Hamacher et al., 2018). In addition, some circulating factors could limit the action of β-adrenergic agonists (Berthiaume et al., 2002).

Corticosteroids are commonly used for both prevention and treatment of ARDS, given their anti-inflammation properties (Khilnani and Hadda, 2011). Although nearly 20% of patients with ARDS receive systemic steroids, there is no clear-cut efficacy of steroids in attenuating lung injury (Boyle et al., 2013; Bellani et al., 2016). Similarly, non-steroidal anti-inflammatory agents ketoconazole and lisofylline also failed in clinical trials for the early treatment of ARDS (The ARDS Network, 2000; The ARDS Clinical Trials Network, 2002). However, the recent identification of ARDS sub-phenotypes (hypo- vs. hyper-inflammatory phenotypes) may help to specify ARDS cohorts and show promise for anti-inflammation therapy to ARDS. For example, statins have been proposed for use in ARDS because of their anti-inflammatory function beyond lowering cholesterol, and they have shown to significantly improve the 28-day survival of patients compared to placebo in a “hyper-inflammatory” subgroup (Calfee et al., 2018). However, no difference was detected in a “hypo-inflammatory” subgroup in terms of the same outcome. Nitric oxide (NO) is crucial in regulating vascular tone and blood flow. Inhaled NO has also been shown to improve pulmonary gas exchange and oxygenation in animal models of ARDS, but as for most other drugs, in patients, inhaled NO has shown no long-term survival benefits (Putensen et al., 1994; Rossaint et al., 1995; Adhikari et al., 2007).

N-Acetylcysteine (NAC) is a common antioxidant widely used for treating conditions characterized by the generation of free oxygen radicals (Shahin et al., 2009). It has been tested in multiple trials on sepsis and lung-injury related ARDS, as well as COVID-19-induced ARDS. However, the benefit of antioxidant therapy with NAC is not consistent among studies, including early mortality rate, duration of ICU stay, and oxygenation (Adhikari et al., 2004; Shahin et al., 2009; Lu et al., 2019). A recent clinical study using NAC for COVID-19 treatment, a high dose of NAC showed no significant benefit in terms of mortality, ICU admission, or time of invasive mechanical ventilation compared to the placebo group (de Alencar et al., 2020).

Apart from these examples, a number of potential therapies have shown promising results in preclinical studies but have been proven ineffective or even harmful in clinical trials. These include surfactant replacement, neutrophil elastase (NE) inhibitors, aspirin, heparin, and angiotensin-converting enzyme (ACE) inhibitors. The use of ACE inhibitors has been limited in preclinical studies because of their side effects on systemic hypotension (Arndt et al., 2006). However, ACE2, which counteracts the activity of ACE, was demonstrated to have a treatment effect on patients with ARDS in a pilot study (Imai et al., 2005; Khan et al., 2017). In addition, ACE2 is a functional receptor for SARS-CoV-2, the coronavirus that caused the COVID-19 pandemic in 2020, and recombinant ACE2 has entered clinical trials (NCT04335136) for COVID-19 (Bao et al., 2020).

## Pulmonary Structural Barriers for Gene Delivery

The lung is a highly specialized and delicate organ that has evolved to maximumly expose blood to air for gas exchange (Hsia et al., 2016). Functionally, conducting airways connecting the inner and outer pulmonary environments dominate the airflow during inhalation and exhalation. Thus, easy access to both the airways and vascular network makes the lung attractive for gene delivery (Pouton and Seymour, 2001; Gautam et al., 2002). Genes can be easily administered through intranasal or oral inhalation using nebulizers or by bronchoscopy-mediated intratracheal administration (Gautam et al., 2002; Katz et al., 2019). For small rodents, oropharyngeal aspiration is a straightforward and simple method for gene delivery into the lung (Zhang S. et al., 2013; Bale et al., 2016; Lin et al., 2016). Compared to intravascular injection, airway delivery shows lower DNA and RNA degradation by nuclease activities (Liu et al., 2007; Henning et al., 2010). Normally, the human lung has approximately 480 million alveoli, which compose a large surface area (∼140 m; Guggino and Cebotaru, 2017) for any gene delivery and account for more than 99% of total lung internal surface area (Crapo et al., 1983; Ochs et al., 2004). Furthermore, there is a massive pulmonary micro-capillary network surrounding alveoli for gas exchange. The thin alveolar epithelial monolayer (0.1–0.5 μm; Weibel, 1973) and its formed massive lung surface area, together with the high permeability of the alveolar-capillary membrane, provide superior conditions for gene and/or particle deposition and uptake into the lung (Labiris and Dolovich, 2003).

Although the lung is unique and has advantages for gene delivery, low transfection efficiency has hindered the progress of gene therapy for lung diseases because of multiple barriers, such as complex branching of the conducting airways, alveolar-capillary barrier, surfactant, lining fluid and mucus, basement membrane, and host immunological defenses. The conducting airways defend the lungs from exogenous particles and bacterial and viral insults through mucociliary clearance (Nicod, 2005). However, they also trap therapeutic genes and decrease the efficiency of delivery (Duncan et al., 2016; Bustamante-Marin and Ostrowski, 2017). For patients with cystic fibrosis, their airways are progressively filled with thickened mucus that forms a solid barrier hindering viral vector or nonviral liposomal vector penetration into the lungs (Sanders et al., 2009; Schuster et al., 2014). Sufficient delivery of gene materials into the parenchymal session of the lungs is also challenging since it heavily relies on particle deposition (Paranjpe and Müller-Goymann, 2014). The respiratory airway branches multiple times to the distal terminal bronchioles and ends in the alveolar sacs (Patwa and Shah, 2015; Hsia et al., 2016; Sondhi et al., 2017). Once delivered cargos, e.g., plasmids, viral vectors, peptides, and siRNA, reach the alveoli, they face the risk of phagocytosis by resident alveolar macrophages (Patton, 1996; Patton et al., 2004; Katz et al., 2019). Localization of a Cy3-labeled adenoviral vector by fluorescence microscopy shows that adenoviral vectors are rapidly internalized (∼1 min) by alveolar macrophages after reaching the alveolar surface (Zsengellér et al., 2000). In a mouse model, within 24 h following administration, 70–90% of adenovirus genomes were cleared, indicating the significance of macrophages in transfection efficiency (Worgall et al., 1997). Surfactant proteins, SP-A and SP-D, can interact with gene delivery agents containing carbohydrate domains, decreasing their transfection efficiency (Vadolas et al., 2002). In addition, the thin alveolar epithelial monolayer provides a large surface area for particle deposition (Labiris and Dolovich, 2003); meanwhile, the tightness formed by junction molecules between cells increases difficulties for gene transfer to subepithelial cells (Patton and Byron, 2007; Murgia et al., 2014).

In addition, the pathological conditions of several diseases would complicate those barrier mechanisms, and metabolic products could also be potential barriers hindering gene delivery (Weiss, 2002). For example, sputum from patients with CF could slow down the adeno-associated virus (AAV) vector diffusion rate by >1,000 fold compared to water (Schuster et al., 2014), and some patients’ sputum also contains adenovirus-specific antibodies that neutralize the Ad vectors for further inhibition (Perricone et al., 2000). During acute lung injury and ARDS, pulmonary edema influx, which results from the loss of the alveolar epithelium and alveolar capillary barrier dysfunction, collapsed alveoli, excessive mucus secretion, and the proinflammatory environment make it hard to transduce the injured lung (Zhang et al., 1998; Weiss, 2002; Matthay et al., 2019).

## Gene Delivery Systems

Because both the cell membrane and nucleic acids are highly negatively charged and, thus, repel each other, delivering exogenous genetic materials into cells requires either a carrier or vector to mask the nucleic acid’s charge or a physical method to circumvent the membrane. Vectors usually refer to virus particles, and carriers typically imply nonviral chemical agents. Ideally, the vector/carrier should show high transduction efficiency with a sufficient number of cells transduced, cell or tissue specificity, enough stability to protect the transgene from extracellular and intracellular degradation, and, perhaps most importantly, minimal immune and inflammatory responses (Mehier-Humbert and Guy, 2005; Katz et al., 2013, 2019). Gene delivery vectors can be classified into two broad categories: viral vectors and nonviral physical or chemical methods (Nishikawa and Huang, 2001; Nayerossadat et al., 2012; Ginn et al., 2018). In either case, high-quality, clinical-grade vectors (viral or nonviral) could be used for any clinical trial. Currently, a number of biotechnology and pharmaceutical companies are focusing on the development of virus- and nonvirus-based systems for gene therapy with the goal of providing single-dose medications available for use in the ICU or even in the outpatient setting (depending on the indication). Thus, like the mRNA-based Covid19 vaccine, any nucleic acid-based gene therapy approach would be available from hospital pharmacies for direct use in patients.

### Viral Vectors

Viral vectors are widely used for gene therapy because of their high transduction efficiency and oftentimes long duration of transgene expression (Bouard et al., 2009). For use as a gene transfer vector, the viral genome is modified to limit viral replication by removing critical viral genes, for example, Gag, Pol, and Env genes in retroviruses, and replacing them with the desired transgene for the target protein (Verma and Weitzman, 2005). Both RNA and DNA viruses have been employed extensively for lung gene delivery (Driskell and Engelhardt, 2003; Sondhi et al., 2017). In the case of RNA viruses, the retrovirus was first used in *ex vivo* lung gene therapy for α1AT deficiency (Garver et al., 1987). Because the RNA genome requires conversion into double-stranded DNA, which then can integrate into the host chromosomal genome (Chiu and Davies, 2004; Yi et al., 2011; Sondhi et al., 2017), it was seen as an ideal vector for long-term durable expression. However, retroviruses are only able to transduce proliferating cells (Hu and Pathak, 2000; Schambach and Morgan, 2016) and have been of limited use for *in vivo* lung gene delivery, since most of the cells in the lungs are not dividing at any given time (Driskell and Engelhardt, 2003; Sondhi et al., 2017). Compared to retroviruses, lentiviral vectors are able to infect nondividing cells (Naldini et al., 1996)and have been proven to be more useful for lung gene delivery (Driskell and Engelhardt, 2003; Patel M. et al., 2013; Marquez Loza et al., 2019). Two types of lentivirus, human immunodeficiency virus (HIV; Goldman et al., 1997) and feline immunodeficiency virus (FIV; Wang et al., 1999), have been shown to infect the airway epithelium for cystic fibrosis gene therapy. However, their application is hindered by the lack of suitable receptors expressed on the epithelial apical surface for viral approach (Walters et al., 1999). In the face of this disadvantage, envelope glycoprotein-pseudotyped lentiviruses were developed to widen their host range (James et al., 2005). For example, pseudotyped lentiviral vectors from filoviruses (Kobinger et al., 2001; Sinn et al., 2017a), baculovirus (Sinn et al., 2012, 2017b), Ebolavirus (Kobinger et al., 2001), influenza virus (Patel M. et al., 2013), and Sendai virus (Mitomo et al., 2010; Griesenbach et al., 2012) all confer access to receptors on the apical side of airway epithelial cells, allowing gene transfer to these cells. In contrast, vesicular stomatitis virus (VSV) glycoprotein-pseudotyped vectors predominantly enter from the epithelial basolateral surface (Goldman et al., 1997; Johnson et al., 2000; Kremer et al., 2007), which limits their direct translation into clinical use. Since RNA viruses integrate the transgene into the host genome in a random fashion, there exists the possibility of oncogene activation or induced mutagenesis for these vectors (Anson, 2004; Bushman, 2007; Milone and O’Doherty, 2018). So far, most application of lentivirus to the lung has focused on chronic diseases, e.g., cystic fibrosis, rather than ARDS, and it is being used for RNA interference-mediated gene knockdown (Tiscornia et al., 2003; Copreni et al., 2004). One study using lentivirus delivered shRNA to silence CD36, which is required for latent TGF-β1 activation, and showed antifibrotic effects after injury to the lung in a silicosis model (Wang et al., 2009). However, since short-term gene expression is more desirable for acute indications like ARDS, such integrating vectors are not appropriate.

The most studied and widely used DNA viral vectors include replication-deficient adenovirus (Ad) and adeno-associated virus (AAV) derived vectors (Crystal, 2014; Katz et al., 2019). Ad contains a large, double-stranded linear DNA genome (∼36 kb), whereas AAV contains a single-stranded DNA genome that is relatively small (∼4.7 kb) (Verma and Weitzman, 2005). Since the Ad genome can be transcribed and replicated episomally (Samulski and Muzyczka, 2014; Sondhi et al., 2017), without necessary integration into the host genome, Ad vectors can confer moderate duration expression and show high transduction efficiency in non-dividing cells of the airway (Crystal, 2014). Unfortunately, Ad vectors are highly immunogenic and induce strong host inflammatory and immune responses specifically against products of these viral genes (Schiedner et al., 1998; Ahi et al., 2011; Lundstrom, 2018), which hinder repetitive administration of the vector and limit gene expression to 2–3 weeks (Crystal, 2014). Several generations of Ad vectors have been developed in order to minimize the host inflammatory and immune responses (Capasso et al., 2014). Compared to the first and second generations of Ad vectors with partial viral genome deletion (Verma and Weitzman, 2005; Capasso et al., 2014), the third generation, called “gutted/helper dependent” Ad vector (Kochanek et al., 2001; Capasso et al., 2014), has had the whole viral coding region deleted to minimize viral antigen expression (Alba et al., 2005; Verma and Weitzman, 2005). Although these advanced Ad vectors enable *in vivo* gene delivery by maximally reducing initial inflammatory responses to administration (Koehler et al., 2006), their capsid proteins can still induce cytotoxic T-cell destruction of the infected cells by antigen presentation and induce antibody production, again preventing subsequent vector administration (Alba et al., 2005; Katz et al., 2019). Of note, a common drawback to *in vivo* viral delivery is immune responses that might confront animals or patients with preexisting lung injuries to a higher risk of inflammatory toxicities (Lin and Dean, 2011). For example, adenovirus transfected rat lungs with high efficiency and uniform distribution of marker genes; however, all end point measurements, such as AFC, were taken 7 days after animal recovery because of viral infection-induced inflammatory responses (Factor et al., 1998). In comparison, electroporation is an effective method to deliver plasmid DNA to living animal lungs with no extra damage and high-level gene expression (Somiari et al., 2000; Dean et al., 2003).

Compared to Ad vectors, AAV vectors show less immunogenicity but have high transduction efficiency, persistent transgene expression, and broad host range (Mingozzi and High, 2013; Samulski and Muzyczka, 2014). Several serotypes of AAV vectors, for example, 2, 5, 6, and 9, have been shown to effectively transduce the airway and alveolar epithelium, and have allowed limited re-administration in experimental animals (Limberis et al., 2009; Li et al., 2011). In a rat model of ARDS, delivery of aerosolized AAV serotypes 2 and 6 to rat lung showed high transduction efficiency of transgene expression, which significantly prevented subsequent LPS-induced lung injury in a protection model (MacLoughlin et al., 2015). AAV-mediated gene therapy for CF has even moved into phase I/II clinical trials (Guggino and Cebotaru, 2017). However, the application of AAV for clinical trials is still limited because of its small packaging capacity, difficulty producing large quantities, issues with re-dosing, and various immune responses in different organs (Mingozzi and High, 2011; Sondhi et al., 2017; Wang et al., 2019).

### Nonviral Gene Delivery: Chemical Vectors

Although viral vectors remain the major delivery method for gene therapy, accounting for approximately two-thirds of total vectors used in clinical trials in 2017 (Ginn et al., 2018), nonviral vectors have been increasingly used in clinical trials since 2004 (∼23%) (Ginn et al., 2018). Compared to viral vectors, nonviral vectors possess some inherent advantages for gene delivery (Nayerossadat et al., 2012; Yin et al., 2014; Ginn et al., 2018; Patil et al., 2019): (1) much larger transgene packaging capacity; (2) much better safety profile; (3) ability to carry and deliver DNA or RNA by chemical carriers; (4) ability for repeat administration; (5) low immunogenicity due to lack of antigen presentation to adaptive immune system; and (6) ease of synthesis and production in large quantities. Indeed, DNA- and RNA-based vectors (plasmids, minicircles, mRNA, and siRNA) are simple and relatively inexpensive to produce on a large scale, especially when compared to their viral counterparts. Although nonviral gene transfer has been widely performed in research in laboratories, its applications in clinical trials have been hindered by several obstacles, such as lower transfection efficiency, lack of specific cell targeting, and lack of stability compared to viral vectors (Glover et al., 2005; Ramamoorth and Narvekar, 2015; Ginn et al., 2018). For example, naked plasmid DNA delivered systemically is degraded quickly, and its half-life is estimated to be only 10 min following intravenous (IV) injection in mice (Kawabata et al., 1995). Thus, various physical and chemical methods have been developed to enhance gene transfection efficiency *in vivo* (Nayerossadat et al., 2012). Cationic lipid- (lipoplexes) and cationic polymer- (polyplexes) based vectors are the most commonly used chemical transfection reagents and are extensively used in gene transfer to the lungs (Davis and Cooper, 2007; Aneja et al., 2009; Jones et al., 2013). Both types of delivery agent interact electrostatically with the negatively charged DNA, forming a net positively charged lipoplex- or polyplex-DNA complex for further interaction with the cell membrane (Zuidam and Barenholz, 1998; Zhu and Mahato, 2010; Jones et al., 2013). Consistent with fundamental purposes of using vectors for gene delivery, namely to overcome multiple extracellular and intracellular barriers and facilitate therapeutic nucleic acids reaching target cells or tissues, chemical vectors are designed to increase the stability and transfection efficiency of DNA complexes and decrease their biodegradability (Zhu and Mahato, 2010). Following intravenous administration, DNA complexes face multiple barriers, such as endonuclease degradation, traversing the vascular wall, intercellular junctions, the cytoplasmic membrane of target cells, and avoiding entrapment in endosomal vesicles (Song et al., 1997; Pack et al., 2005; Hill et al., 2016). Thus, it is important to investigate vector structure-activity relationships and their optimization for lung gene delivery.

Cationic lipids are diverse in structure, but there are three basic structural components, a cationic head group, a hydrophobic tail, and a linker connecting both the head and tail groups (Zhu and Mahato, 2010; Jones et al., 2013). The positively charged hydrophilic head group is the major domain that interacts with negatively charged DNA molecules, leading to plasmid condensation, enhanced cellular uptake, and endosomal escape (Miller et al., 1998; Tseng et al., 2009; Zhu and Mahato, 2010). The hydrophobic tail group is usually composed of saturated or monounsaturated fatty acid chains (aliphatic chains) with various lengths (Niculescu-Duvaz et al., 2003; Kou et al., 2011). It is widely accepted that gene transfection efficiency is inversely correlated with chain length, which means the shorter the chain length (e.g., C14), the higher the transfection efficiency. This is presumably due to increased lipoplex stability (Felgner et al., 1994; Adir et al., 2008; Jones et al., 2013). Cholesterol is a commonly used alternative for the hydrophobic tail domain and shows enhanced fusion with the host cell membrane (Mahato et al., 1997; Jones et al., 2013; Monteiro et al., 2014). The linker that connects the cationic head to the hydrophobic tail domain can greatly impact the stability and biodegradability of the lipoplex (Mahato et al., 1999). Ether bonds, such as those in DOTMA, are indicated to render good gene delivery efficiency because of their stable and nondegradable properties, but their cytotoxicity is higher than that of other linker chemicals, such as esters and amides (Singhal and Huang, 1994; Freedland et al., 1996; Zhu and Mahato, 2010; Jones et al., 2013). Cleavable linkers are also used as an alternative. Additionally, the cationic nature of the head group can be another source of cytotoxicity, since it can interact non-specifically with negatively charged serum proteins (albumin, lipoproteins, and IgG), resulting in charge neutralization, reduced cellular uptake, hemolysis, and decreased transfection efficiency (Escriou et al., 1998; Zelphati et al., 1998; Lv et al., 2006; Jones et al., 2013). It has been shown that intravenous injection of DNA lipoplexes actually induces embolization in the lungs because of large complex size (>5 μm) by interaction with blood components, leading to failure to pass through capillaries (Litzinger et al., 1996; Nishikawa and Huang, 2001).

Cationic polymers also form complexes with DNA through electrostatic interactions and coat the complex with a net positive charge (Nishikawa and Huang, 2001; Eliyahu et al., 2005). Polyethylenimine (PEI) is one of the most commonly used synthetic polymers with a highly positive charge (Eliyahu et al., 2005; Jin L. et al., 2014). The different amine groups in PEI’s structure affect the polyplex’s endosomal escape after uptake by displaying buffering capacities over a wide range of pH (Zhu and Mahato, 2010). It has been proposed that different types of amines work as a “proton sponge”, which can be protonated to different levels as the endosomal environment acidifies, leading to ultimate endosomal breakup and release of their contents (i.e., DNA) (Boussif et al., 1995; Eliyahu et al., 2005; Pack et al., 2005; Jin L. et al., 2014). Compared to linear PEI, which contains almost all secondary amines in its backbone, branched PEI contains primary, secondary, and tertiary amino groups (Fischer et al., 1999; Zhu and Mahato, 2010), which confers PEI with larger buffering capacity (Boussif et al., 1995), leading to early endosomal escape of plasmids, and offers protection of the DNA from lysosomal degradation (Zhu and Mahato, 2010). In PEI, the nitrogen to DNA phosphate (N/P) ratio, one indicator of the properties of DNA polyplex, along with complex size, net surface charge, and stability, is associated with transfection efficiency (Nimesh et al., 2007; Vu et al., 2012; Gary et al., 2013). With an N/P ratio < 1, the PEI/DNA complex is characterized by incomplete DNA condensation, whereas when the ratio > 3, the free PEI is thought to enhance endosomal escape, contributing to DNA intracellular release (Mislick and Baldeschwieler, 1996; Boeckle et al., 2004; Perevyazko et al., 2012; Jin L. et al., 2014). The molecular weight of PEI is another determinant of transfection efficiency (Jones et al., 2013; Jin L. et al., 2014).

### Physical Methods for Gene Delivery

Gene transfer of naked or plasmid DNA by physical means is an attractive delivery system for gene therapy because it is simple and has low cytotoxicity (Gao et al., 2007). Physical delivery approaches are popular in clinical trials, accounting for 14% in 2004, 18% in 2007, 18.3% in 2012, and 16.6% in 2017, of total gene therapy clinical trials (Edelstein et al., 2004, 2007; Ginn et al., 2013, 2018). Physical methods to introduce exogenous genes into cells have been explored both *in vitro* and *in vivo* (Dean et al., 2003; Gehl, 2003; Matsuda and Cepko, 2004; Dean, 2005). Basically, a physical force, produced by mechanical force, electrical pulses, ultrasound, laser irradiation, or magnetic fields, is employed to transiently disrupt the cell membrane and create small pores, so that DNA can diffuse into cells (Schneckenburger et al., 2002; Gehl, 2003; Mehier-Humbert and Guy, 2005; Li et al., 2008; Liu et al., 2012; Nayerossadat et al., 2012). Although naked DNA can be directly injected into local tissues, e.g., skeletal muscles or liver, and into the systemic circulation *via* tail vein, this “simplest” delivery method shows low transfection efficiency due to rapid *in vivo* degradation by nucleases and clearance by tissue-resident macrophage (e.g., Kupffer cells in the liver), limited extravasation from the circulation, and high interindividual variability (Kawabata et al., 1995; Mahato et al., 1995; Mir et al., 1999; Heller et al., 2000; Zhang et al., 2012; Yin et al., 2014). Plasmid DNA incubated in isolated rat plasma can degrade quickly with a half-life of 1.2 min for the supercoiled form, 21 min for the open circular plasmid DNA, and 11 min for the linear form (Houk et al., 1999). Cytoplasmic nucleases are another barrier impeding the efficient expression of plasmid DNA (Lechardeur et al., 1999; Bai et al., 2017). In contrast to direct injection of naked DNA into tissues, the “gene gun” or gene-mediated particle bombardment takes advantage of the high velocity of a particle carrier (e.g., gold beads) to deliver DNA into target tissues, such as skin, liver, and muscle (Wolff et al., 1992; Nishikawa and Huang, 2001). This method demonstrates increased transfection efficiency, for example, for epidermal tissues with 10–20% transfection of cells in the bombarded area, but is still limited in clinical trials because of concerns of poor penetration (<0.5 mm depth) into organs (Yang et al., 1990; Williams et al., 1991; Zelenin et al., 1997). The major application of gene gun in human trials is for DNA vaccination or suicide gene therapy to treat cancers (Trimble et al., 2003; Fuller et al., 2006). To date, this approach has not been used successfully in the lungs.

Electroporation, or EP, has been widely used in clinical settings to treat cancer (electrochemotherapy) and deliver drugs or vaccines to target cells. It was first used for DNA transfection of cultured mouse lyoma cells in 1982 (Neumann et al., 1982). It is a fast and reproducible approach, and requires a relatively low dose of DNA (Dean et al., 2003). In principle, when the transmembrane potential applied by the external electric field exceeds the cell resting potential, EP transiently disrupts the cell membrane and forms hydrophilic pores so that various molecules surrounding cell surface, such as DNA, RNA, oligonucleotides, ions, drugs and antibodies, can pass into the cells (Weaver and Chizmadzhev, 1996; Somiari et al., 2000). This delivery approach is not limited to small DNAs like AAV or other viruses. Indeed, delivery of plasmids with large loading capacity (e.g., 100 kb) and co-transfection of several plasmids to cells can be achieved through EP (Magin-Lachmann et al., 2004). For *in vivo* applications, DNA is delivered to the tissue, usually by injection, and then the electric field is applied with penetrating needles or surface electrodes. Several advantages are highlighted for EP in gene transfer *in vivo*. First, EP shows high transfection efficiency with relatively little interindividual variability and increases tissue transgene expression by 100–1,000 folds compared to direct injection of naked DNA. For example, EP of plasmid-encoding IL-5 into mouse tibialis muscle produced 20 ng/ml of IL-5, while direct delivery of plasmids without EP generated only 0.2 ng/ml of IL-5 in the blood (Aihara and Miyazaki, 1998; Mir et al., 1999; Wells, 2004). One critical step for gene transfer by EP is that EP should be applied immediately after DNA administration (Gao et al., 2007). For one thing, the short time interval between these two procedures would minimize DNA degradation by extracellular nucleases (Gao et al., 2007). For another, several studies indicate that there is almost no gene transfection, comparable to direct injection of plasmids, if naked DNA is injected into tissue (e.g., skeletal muscle) after EP application, suggesting that DNA must be present while the electric pulse is being applied (Mir et al., 1999; Satkauskas et al., 2002). Second, EP can be used to deliver genes locally to tissues, rather than by systemic delivery, which avoids the unnecessary exposure of other tissues to electric fields and also reduces the DNA dose needed (Mir et al., 1991; Gilbert et al., 1997; Gehl, 2003; Mir, 2014). Any solid tissue, for example, skin, liver, skeletal muscle, lung, kidney, cornea, and retina, prone to exposure to electric fields could be subject to EP-mediated gene delivery (Mir et al., 1999; Blair-Parks et al., 2002; Dean, 2003; Dean et al., 2003; Franquesa et al., 2005; Jaichandran et al., 2006; Zhou and Dean, 2007; Matsuda and Cepko, 2008; Medi and Singh, 2008). The electrodes delineate the area for gene transfer, which increases gene targeting specificity (Gehl, 2003). Third, EP can be applied to all cell types and cells in the dividing and non-dividing stages, since the mechanism of transfection does not depend on the uptake function of cells, but rather the transiently formed pores on the plasma membrane and the electrophoretic force during EP (Wells, 2004; Hirao et al., 2008; Escobar-Chávez et al., 2009). Additionally, EP does not induce any immune response, which is a significant safety concern in viral vector delivery system. However, a recent report indicated that a small transient increase in neutrophils could be detected in the lungs of mice within the first hour of electroporation, but that this returned to normal within 24 h; whether this has any lasting effects is unknown at this point, but, given that EP has been used by multiple groups to treat ARDS in mouse and pig models, suggests that this is not of a great concern (Eliseeva et al., 2021). Some inflammatory responses can be potentially provoked by any unmethylated CpG motifs in plasmids, but this can be reduced by plasmid modification (Krieg et al., 1995; Nishikawa and Huang, 2001).

Electroporation has been developed to deliver plasmid DNA into the lungs to treat diseases (Dean et al., 2003; Hasson et al., 2005; Jones et al., 2005). Traditionally, solid tissues (e.g., skeletal muscle, heart, and liver) have been directly injected with plasmids, and then the electric field is applied for *in vivo* gene delivery (Dean, 2005). However, lungs are not completely amenable to this approach (Dean, 2003; Young et al., 2014). For one thing, plasmid solution usually needs to be injected through some syringe or needle into solid tissues (Wolff et al., 1990), whereas the structure of the lungs is not appropriate for direct injection. For another, the electrodes designed for solid tissues, such as penetrating electrodes and caliper plate electrodes (Somiari et al., 2000), are not suitable for lungs. The lung is a delicate organ, which directly contacts the external environment through the airway and has a large epithelial surface area for gas exchange (Katz et al., 2019). This easy access *via* the airways makes the lung amenable to plasmid delivery through intratracheal administration (Zhou R. et al., 2008). Our laboratory and others have developed protocols for DNA delivery into the lungs (i.e., aspiration or inhalation) followed by EP, which show high transfection efficiency (Dean et al., 2003; Jones et al., 2005; Machado-Aranda et al., 2005; Gazdhar et al., 2006). Specifically, a plasmid solution containing 140 mM NaCl is administered to the lungs by aspiration in anesthetized mice, and then a pair of pre-gelled pediatric pacemaker surface electrodes is placed on either side of the chest under the armpits to deliver electric pulses that would travel through multiple tissue layers, e.g., skin, fat, and muscle, to reach the lungs (Dean, 2003). The parameters for optimal field strength have been determined to be 200 V/cm, using eight continuous 10 ms square wave pulses with 1 s interval (Dean et al., 2003). These parameters are also optimal in rats for lung delivery (Machado-Aranda et al., 2005). For larger animals such as 50-kg pigs, the DNA is delivered to anesthetized animals by bronchoscope to the desired lobe(s), and surface electrodes (in this case defibrillation pads) are used to deliver a train of eight pulses of ∼150 V/cm but with a shorter duration (∼100 to 150 μs each) (Dean et al., 2011; Emr et al., 2015). The distribution of transgene expression in the lungs has been evaluated by transferring reporter genes such as lacZ and GFP (Dean et al., 1999, 2003; Dean, 2003; Gottfried et al., 2016; Lin et al., 2016). Histological, immunohistochemical, and immunofluorescent analyses of mouse lung sections indicate that most cells receive and express transgenes throughout the lungs and in all cell types, such as the airway epithelium, alveolar epithelium (both ATI and ATII cells), endothelial cells, smooth muscle cells (both airway and vascular), and fibroblasts (Dean et al., 2003). Although plasmids could be administered intravenously to target the lungs, DNA nucleases are much higher in the serum than in the airway, and the injected DNA would be quickly degraded in the blood, resulting in low transfection efficiency (Song et al., 1997; Barron et al., 1999).

### Choice of Gene Therapy Approach

Apart from their various properties, the choice of which type of gene therapy vector to use depends in great part on the disease being treated and its presentation. For example, in the case of a monogenetic disease such as sickle cell disease or cystic fibrosis, replacement of the defective genomic copy of the gene may be desired. If this is the case, homologous recombination methods such as CRISPR/Cas9 may be used, delivered either virally or by plasmid. Alternatively, the long-term expression of wild type copies of these genes may be sufficient to overcome the phenotype of the disease (e.g., overexpression of fetal hemoglobin in sickle cell patients), in which case using a viral vector that integrates into the genome such as a retrovirus or lentivirus would be desired. However, in the case of ARDS, three main issues should be considered. First, this is an acute disease that requires limited term expression of transferred genes. For example, if treatment involves overexpressing a Na^+^ transporter such as ENaC or the Na^+^, K^+^-ATPase to reduce pulmonary edema, overexpression should be only for a short time so that disease-associated edema is cleared. If the gene was expressed long-term (e.g., by integration of the vector) in healthy individuals after resolution, increased fluid clearance from the lungs could result in mucus-rich, dehydrated lungs. Second, since ARDS is an acute-onset disease, any gene that is transferred to the lungs should be turned on quickly so that it would have maximal time to elicit benefit. In this case, mRNA could be a great choice, since it leads to almost immediate translation of proteins upon entry into the cell. However, the only drawback to mRNA approaches is that they are transient (perhaps too transient in this case), and unstable since mRNA is rapidly degraded by host nucleases. Plasmid DNA can also elicit rapid gene expression following entry into cells and tissues, with significant levels of expression seen in skeletal muscle in mice being detected within minutes of injection (Doh et al., 1997). Finally, in inflammatory diseases such as ARDS, the last thing that is wanted is using a gene delivery system that exacerbates the injury by causing more inflammation. Thus, viral vectors are not the best choice; nonviral vectors, with their greater safety profile, would be a more appropriate choice.

### Current Gene Therapy for Acute Respiratory Distress Syndrome

**FIGURE 3:**
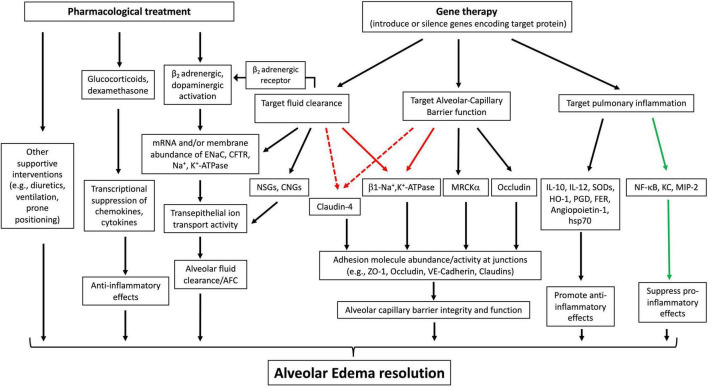
Therapeutic approaches to treat acute lung injury (ALI)/ARDS. Pharmacological interventions primarily focus on enhancing fluid clearance through b2 adrenergic or dopaminergic activation, dampening inflammation with glucocorticoids, and use of supportive strategies, all of which have led to limited therapeutic effects. Gene therapies to overexpress or silence target genes have targeted alveolar fluid clearance, alveolar capillary barrier function, and pulmonary inflammation. The protein targets of gene therapy for ARDS/ALI have been validated in various animal or *ex vivo* organ models, indicating their therapeutic potential. Black solid arrow: increase, upregulate or target downstream effects; green solid arrow: decrease or inhibit target proteins; red solid arrow: has been validated in various animal models to have a dual role in both upregulating fluid clearance and lung barrier function; red dotted arrow: has the potential for dually upregulating fluid clearance and lung barrier integrity. ENaC, epithelial sodium channel; CFTR, cystic fibrosis transmembrane conductance regulator; NSGs, non-selective cation channels; CNGs, cyclic nucleotide-gated channels; MRCKa, CDC42 binding protein kinase alpha; ZO-1, zonula occludens-1; VE-cadherin, vascular endothelial-cadherin; HSP-70, heat shock protein 70; HO-1, heme oxygenase-1; KC, murine chemokine CXCL1; MIP2, murine chemokine CXCL2; PGDsyn, prostaglandin D synthase.

A number of gene-based therapies have been developed over the past few decades for ARDS treatment (Table 1). In contrast to hereditary diseases, which require permanent alteration of the defective genome, ARDS is an acute disease of lung dysfunction, so short-lived or transiently altered gene expression is sufficient to treat the disease (Devaney et al., 2011). Gene delivery systems, such as viral vectors and conventional nonviral vectors, and physical delivery approaches have been well- developed to target the lungs for overexpression or silencing and make pulmonary gene transfer clinically possible (Jin L. et al., 2014; Ginn et al., 2018). Unfortunately, ARDS is not caused by a single gene, making gene therapy more difficult. However, a number of obvious target genes exist to promote AFC and edema resolution, repair the alveolar capillary barrier function, and relieve inflammation (Figure 3).

**TABLE 1:** Gene therapy approaches for acute respiratory distress syndrome (ARDS).

Target gene	Vector	Results	Clinical significance	Lung injury model	References
***Gene therapy to enhance AFC***					
ATP1 b1	Ad	Basal AFC, 100%	–	Normal rat without injury	Factor et al., 1998
ATP1 a2	Ad	+Na, K-ATPase activity; AFC	–	Normal rat without injury	Ridge et al., 2003
ATP1 b1	Ad	+AFC +Survival	Prevention	Hyperoxia and rat	Factor et al., 1999, 2000
ATP1 b1	Ad	+AFC, 100%	Prevention	Acutely elevated left atrial pressure	Azzam et al., 2002
b2 Adrenergic receptor	Ad	+AFC, 100% +Receptor function	–	Normal rat without injury	Dumasius et al., 2001
ATP1 a1 and ATP1b1	Cationic lipoplex	-W/D ratio +Na pump activity	Prevention	Thiourea, I.P injection, and mouse	Stern et al., 2000
ATP1 b1	Ad	+AFC	Prevention	High tidal volume ventilation and *ex vivo* rat	Adir et al., 2003
ATP1 a2	Ad	+AFC +Na pump activity -Barrier permeability	Prevention	High tidal volume ventilation and *ex vivo* rat	Adir et al., 2008
ATP1 b1	EP	+AFC, 100%	–	Normal rat without injury	Machado-Aranda et al., 2005
ATP1 b1	EP	+AFC, -W/D ratio -Cellularity, -protein in BAL, -barrier permeability	Prevention; treatment	I.t. delivery of LPS and mouse	Mutlu et al., 2007; Lin et al., 2016
ATP1 b1 ENaC-α1	EP	-W/D ratio, +survival, +Compliance, +PaO2/FiO2 ratio	Treatment	PS + I/R in gut, pig	Emr et al., 2015
CFTR	Ad	AFC, 100%	–	Normal rat without injury	Mutlu et al., 2005
***Gene therapy to target pulmonary inflammation***					
IL-10	Ad	-TNFα, IL-1, MPO in BAL -Inflammation -Histological injury +IL-10 level	Prevention	I.t. delivery of bacteria, pig	Morrison et al., 2000
IL-10	AAV	-Proinflammatory cytokines (IL-1β, TNFα, MIP-1α, and KC) -Histological injury	Prevention	I.t. delivery of bacteria, mouse	Buff et al., 2010
IL-10	Cationic lipoplex	-Lung, liver, and kidney injury (PMNs, MPO, and MDA) -TNFα	Prevention	Cecal ligation and puncture model of sepsis, mouse	Kabay et al., 2007
IL-10	Ad	Dose of adv vector inversely correlates to survival rate	Prevention	I.p. injection of zymosan, mouse	McAuliffe et al., 2006
IL-12	Ad	+Survival +IL-12 level in tissue and BAL	Prevention	I.t. delivery of bacteria, mouse	Greenberger et al., 1996
TGFb1	Cationic lipoplex	-Histologic score of rejection +Oxygenation	Prevention	Acute lung allograft rejection by left rat lung transplantation	Mora et al., 2000
Manganese SOD	Ad	+AFC +Na pump activity	Prevention	Hypoxia and rat	Litvan et al., 2006
CuZn-SOD; catalase	Ad	-Histologic injury score, inflammation +SOD, catalase activity	Prevention	100% oxygen, rat	Danel et al., 1998
Extracellular SOD	AAV	+Oxygen saturation +Lung compliance -PMNs, protein in BAL	Prevention	I.t. delivery of LPS, rat	Hassett et al., 2011
HO-1	Ad	+Survival -PMNs in BAL -Histologic injury	Prevention	Hyperoxia and rat	Otterbein et al., 1999
HO-1	Ad	-Histologic injury -Cytokine (KC, TNFα, and IL-10) in BAL	Prevention	Inhalation of aerolized LPS, mouse	Inoue et al., 2001
HO-1	Ad	-PMNs, KC, and TNFα in BAL -Epithelial cell death	Prevention	I.t. delivery of bacteria, mouse	Tsuburai et al., 2004
HO-1	Ad	+Survival -PMNs, total cell, cytokine in BAL -Histologic injury	Prevention	I.n. delivery of influenza A virus, mouse	Hashiba et al., 2001
HO-1	Cationic polyplex	PMNs, total cell, cytokine in BAL -Histologic injury	Treatment 2 h post	I.t. delivery of LPS, rat, mouse	Kim et al., 2019; Park et al., 2012
Ang-1	Ad	+Survival -Lung barrier permeability	Prevention	Septic shock model by i.p. injection of LPS, mouse	Huang et al., 2008
Ang-1	EP mediated transfection of MSC, *ex vivo*	-Lung barrier permeability (protein, Albumin, and IgM in BAL) -Proinflammatory cytokine, chemokine -Histologic injury	Treatment 30 mins post	I.t. delivery of LPS, mouse	Mei et al., 2007
HSP-70	Ad	-PMN infiltration -Mortality rate -Histologic injury	Prevention	Cecal ligation and double puncture model of sepsis, rat	Weiss et al., 2002
Adiponectin	Cationic polyplex	-TNFα, IL-1β in BAL and tissue -Histologic injury	Prevention	I.t. delivery of LPS, mouse	Piao et al., 2017
PGD synthase	Retroviral infection of fibroblast *ex vivo*	-BAL total cells -Edema +Survival	Prevention	I.t. delivery of bleomycin, mouse	Ando et al., 2003
NF-κB p65	siRNA	-NF-κB, MMP9 -W/D, histology -total cell, IL-6,17 in BAL	Prevention	Cecal ligation and puncture model of sepsis, mouse	Jin L.-Y. et al., 2014
NF-κB	siRNA	-NF-κB, TNFa level -W/D, white blood cell +Rectal temperature	Prevention	I.p. injection of LPS, rat	Li et al., 2016
KC, MIP-1	siRNA	-IL-6, MPO, and MIP-2 KC and PMNs	Prevention	Cecal ligation and puncture after hemorrhage induction, mouse	Lomas-Neira et al., 2005
FER	EP	+Survival, lung compliance, anti-bacterial immune response, and bacterial clearance -Total cells in BAL -Histological injury	Prevention	Lung contusion with secondary bacterial pneumonia, mouse	Dolgachev et al., 2016
FER	EP	+Survival, anti-bacterial immune response (IFN-γ, TNF-α, and KC), bacterial clearance	Prevention Treatment	Bacterial pneumonia and mouse	Dolgachev et al., 2018
***Gene delivery to restore alveolar capillary barrier function***					
ATP1 b1	EP	+AFC, -W/D ratio -Cellularity, -protein in BAL, -barrier permeability	Treatment	I.t. delivery of LPS, mouse	Lin et al., 2016
ATP1 b1	EP	+AFC, -W/D ratio -Cellularity, protein in BAL	Treatment	I.t. delivery of LPS, mouse	Mutlu et al., 2007
ATP1 b1 and ATP1 a2	EP	+Lung compliance -Lung barrier permeability -Histologic injury	Treatment (immediately after trauma)	Lung contusion by blunt chest injury, mouse	Machado-Aranda et al., 2012
MRCKα	EP	-W/D ratio -Cellularity, -protein in BAL, -barrier permeability	Treatment	I.t. delivery of LPS, mouse	Liu and Dean, 2021
FER	EP	+Survival, lung compliance -Lung permeability -Total cells in BAL -Histological injury	Prevention	Lung contusion with secondary bacterial pneumonia, mouse	Dolgachev et al., 2016
Ang-1	Ad	+Survival -Lung barrier permeability -W/D, MPO	Prevention	Septic shock model by i.p. injection of LPS, mouse	Witzenbichler et al., 2005; Huang et al., 2008

*+, increase; –, decrease. ATP1, Na^+^, K^+^-ATPase; W/D, wet to dry ratio; CFTR, cystic fibrosis transmembrane conductance regulator; KC, murine chemokine CXCL1; MIP2, murine chemokine CXCL2; MIP-1a, macrophage inflammatory protein-1 alpha; PMN, neutrophil; MPO, myeloperoxidase; MMP9, matrix metallopeptidase 9; MDA, malondialdehyde; SOD, superoxide dismutase; NOS-2, nitric oxide synthase; PGD synthase, prostaglandin D synthase; HSP-70, heat shock protein 70; HO-1, heme oxygenase-1; MRCKa, CDC42-binding protein kinase alpha; AFC, alveolar fluid clearance; BAL, bronchoalveolar lavage fluid; TEER, transepithelial/trans-endothelial electrical resistance; EP, electroporation.*

### Gene Therapy to Improve Alveolar Fluid Clearance

In the setting of lung injury or ARDS, fluid clearance is impaired, and reduction in AFC rate correlates with increased mortality (Ware and Matthay, 2001; Huppert and Matthay, 2017). Thus, enhancing AFC has been considered one of the primary therapeutic goals for gene therapy for ARDS. Fluid clearance is based on the osmotic pressure created by transepithelial ion transport. Thus, similar to the principle of using β_2_ agonists to enhance fluid clearance, gene delivery to potentiate AFC is based on the rationale that by introducing genes encoding various ion channels or transporters into the lungs, ion transport activities can be directly or indirectly upregulated. However, underlying the complexity of the disease, many factors need to be considered to develop any ARDS gene therapy. First, are the mechanisms known to stimulate AFC in normal lungs also effective in acutely injured lungs? One possible reason accounting for the failure of β_2_ agonist therapy is the desensitization and internalization of β_2_ receptors by long-term endogenous catecholamine stimulation, leading to decreased response during disease. Adenovirus-mediated gene transfer of the β_2_-adrenergic receptor into healthy rat lungs increased receptor sensitivity to endogenous catecholamines and consequently upregulated Na^+^, K^+^-ATPase activity and ENaC expression in the lungs, leading to improved AFC (Dumasius et al., 2001, 2003). In a hyperoxia-induced injury model, gene transfer of the β_2_ receptor protected animal lungs from subsequent injury, showing decreased lung edema and increased survival (Mutlu et al., 2004). However, most studies showing beneficial effects by upregulating β_2_ receptors in lung injury have been limited to animal models with gene delivery prior to inducing injury, and such protection studies have limited clinical significance.

Second, what aspects really matter for the upregulation of fluid clearance by delivered genes encoding different ion transporters or channels? The underlying mechanisms of catecholamine-activated Na^+^ transport include increased protein synthesis and membrane recruitment of the Na^+^, K^+^-ATPase and ENaC, as well as increased open probability of the channels (Yue et al., 1995; Minakata et al., 1998; Bertorello et al., 1999; Saldías et al., 1999). Although catecholamine activation significantly increases edema clearance in various animal models and in isolated human lungs (Sakuma et al., 1994; Lecuona et al., 1999; Frank et al., 2000), the experimental design of these studies is not entirely reflective of the pathophysiology of ARDS in which the alveolar-capillary barrier is at least partially disrupted. In all these experimental cases, there is an intact alveolar epithelial barrier, allowing for effective AFC by upregulating Na^+^ transport (Mutlu and Sznajder, 2004). This brings up the fact that effective net AFC depends on an intact epithelial barrier that can transport ions across the alveolar epithelium (Huppert and Matthay, 2017). Overexpression of Na^+^, K^+^-ATPase, ENaC, cystic fibrosis transmembrane conductance regulator (CFTR), or other ion channels through gene delivery directly increases ion transport activity, accelerating fluid clearance. For example, overexpression of the Na^+^, K^+^-ATPase α2, or β1 subunit through adenoviral transfer or electroporation of β1 subunit plasmids into healthy rat lungs increased fluid clearance rates by >100 (Factor et al., 1998), 250 (Ridge et al., 2003), and 74% (Machado-Aranda et al., 2005), respectively. Adenoviral delivery of the Na^+^, K^+^-ATPase into rat lungs before induction of lung injury (e.g., by hypoxic injury or VILI) prevented and even somewhat reversed the decrease in AFC, compared to injury alone (Factor et al., 2000; Adir et al., 2003, 2008). However, in most *in vivo* studies, adenoviral vectors were delivered either to healthy lungs or prior to induction of lung injury by various insults, such as ventilation, acute elevation of lung pressure, or 100% oxygen, demonstrating that gene therapy to enhance AFC could protect against experimental lung injury. These studies could be implied to suggest that gene delivery to an intact alveolar epithelium was required for upregulating transepithelial ion transport and net AFC. In this respect, the β1 subunit of the Na^+^, K^+^-ATPase has been identified as a unique target for gene delivery to treat acute lung injury, since the Na^+^, K^+^-ATPase is not only the major driver of AFC but is also closely involved in the regulation of epithelial barrier integrity (Rajasekaran and Rajasekaran, 2003; Vadasz et al., 2007). Our laboratory has focused on β1 subunit gene therapy using different animal models, such as mice (Mutlu et al., 2007), rats (Machado-Aranda et al., 2005), and pigs (Emr et al., 2015), and our previously published data have shown that electroporation-mediated gene delivery of the β1 subunit of the Na^+^, K^+^-ATPase can rescue endotoxin pre-injured mouse lungs by both increasing AFC and, more importantly, restoring lung barrier function. These studies indicate that lung barrier integrity is important for gene therapy targeted at increasing net AFC (Lin et al., 2016).

Apart from ion channel transporters, some paracellular tight junction molecules, for example, claudin-4, play roles as regulators of ion transport across the epithelium, showing selective permeability to ions (Brune et al., 2015). For one thing, they are required for maximal epithelial barrier function and are upregulated during epithelial repair (Wray et al., 2009). For another, they are not ion transporters but are closely involved in paracellular ion conductance and have an impact on fluid clearance (Rokkam et al., 2011). Because of this, claudin-4 could be a potential target for gene therapy to increase AFC and barrier integrity. Claudin-4 is highly expressed in both ATI and ATII cells (LaFemina et al., 2010), and staining of human lungs shows a positive correlation of claudin-4 with AFC, indicating that higher claudin-4 expression is associated with higher rates of fluid clearance (Rokkam et al., 2011). It is still not clear whether claudin-4 levels are altered from baseline during injury in human lungs, although its expression was increased during early experimental lung injury.

Nonselective cation channels (NSCs), cyclic nucleotide-gated channels (CNGs), and the CFTR Cl channel are also expressed apically and contribute to the creation of the transepithelial osmotic gradient (Mutlu and Sznajder, 2005). In fluid-filled lungs of mice with genetic KO of acid-sensing ion channel 1 (ASIC1), a nonselective cation channel, fluid clearance was reduced by more than 50% compared to wild type, which is comparable to the AFC reduction seen in mice with ENaC inhibition, indicating that ASIC1 could also be a potential target for gene delivery for AFC (Trac et al., 2017). CFTR is important for maintaining alveolar fluid homeostasis and has been identified contributing to lung fluid reabsorption (Fang et al., 2002). Indeed, overexpression of CFTR by adenoviral infection of mouse lungs increases AFC 90% over that seen following infection with empty vector control (Mutlu et al., 2005).

### Gene Therapy to Target Pulmonary Inflammation

In the early stage of ARDS, the uncontrolled production of cytokines and chemokines initiate proinflammatory responses, which further exacerbates lung injury. Therefore, gene delivery of target proteins to suppress or dampen proinflammatory effects or enhance anti-inflammatory effects has been studied on various experimental models of ARDS and proven successful *in vivo*. One strategy has been to deliver genes to overexpress anti-inflammatory cytokines, anti-oxidant enzymes, antiproteases, and other protective proteins to attenuate inflammation-associated lung injury during ARDS (Liu and Slutsky, 1997). However, most experiments delivered target genes before inducing injury and primarily demonstrated the protection effect with less clinical significance. IL-10 is an anti-inflammatory cytokine and is produced to suppress proinflammatory responses by inhibiting proinflammatory cytokine release, thus limiting the excessive injury induced by inflammation (Couper et al., 2008). Ad delivered IL-10 into animal lungs prior to bacterial infection significantly prevented histological lung injury, release of proinflammatory TNα in BAL, and overall inflammation (Morrison et al., 2000; Buff et al., 2010). Furthermore, in a systematic sepsis model in mice, lipoplex-delivered IL-10 significantly prevented lung and other organ injury (Kabay et al., 2007). However, it was reported that adenoviral-delivered IL-10 protected lungs with improved outcome only when a relatively low dose of vector was administered and that survival rates actually worsened at higher doses (McAuliffe et al., 2006). This highlights the inherent problems associated with using a proinflammatory viral vector to treat an inflammatory disease. Other cytokine therapeutic targets for gene delivery have included IL-12, which has shown protection from subsequent lethal doses of Klebsiella in a pneumonia model (Greenberger et al., 1996).

Superoxide dismutases (SODs) are a group of metalloenzymes that form the front line of defense against oxidative stress, e.g., ROS, in the body (Landis and Tower, 2005). During ARDS, the overwhelmed inflammatory response leads to excessive ROS, contributing to disease progression (Ware and Matthay, 2000). Overexpression of SOD in transgenic mice has been shown to have a protective effect on lung injury induced by hyperoxia (Ahmed et al., 2003), hypoxia (Litvan et al., 2006), LPS (Bowler et al., 2004), and influenza virus (Suliman et al., 2001). However, the pharmacological administration of SODs for clinical treatment of ARDS has shown limited success due to the enzyme’s short half-life in circulation, rapid renal excretion, and inability to penetrate cells to remove ROS, leading to low accumulation within injured lungs (Danel et al., 1998). Adenoviral-mediated gene transfer of SODs prevented hypoxia-induced lung injury, counteracting ROS-induced endocytosis of the Na^+^,K^+^-ATPase and subsequent decrease in alveolar fluid reabsorption (Litvan et al., 2006). Another study further demonstrated the protection effects of SODs on LPS-induced injury, in terms of physiological oxygen saturation and lung compliance, lung barrier function, and inflammation (Hassett et al., 2011). However, again, all studies have used protection, not treatment protocols, thus calling into question the utility to treat ARDS clinically.

Heme oxygenase-1 (HO-1) is another candidate for gene delivery to fight against excessive oxidative stress during lung injury. It is a critical enzyme for catabolizing heme and shows antioxidant, anti-inflammatory, and anti-apoptotic properties for vascular protection (Araujo et al., 2012). Chemical inhibition or genetic silencing of HO-1 significantly increases proinflammatory cytokine release, immune cell infiltration, and apoptosis, indicating that HO-1 has a protective role in LPS-induced ALI (Wiesel et al., 2000; Gong et al., 2008; Zhang Y. et al., 2013). Ad transfer of HO-1 has been shown to confer protection against hyperoxia, aerosolized endotoxin, and bacteria- or virus-induced lung injury (Otterbein et al., 1999; Hashiba et al., 2001; Inoue et al., 2001; Tsuburai et al., 2004). In a more recent study, the treatment effect of HO-1 nonviral gene delivery was tested, in which HO-1 plasmids were co-delivered *via* a polymeric complex with an anti-inflammatory compound after LPS had been administered intratracheally to induce injury. Thus, gene delivery was carried out in lungs that were already injured. From the results, the histological lung injury and proinflammatory cytokine release were significantly rescued (Kim et al., 2019). Other gene therapeutic targets for anti-inflammatory strategy include angiopoietin-1 (see below), heat shock protein 70, and adiponectin, but while all have shown some promise in limiting inflammation, improving edema resolution, and/or improving survival, all have either carried out protection studies only, or have failed to have an effect when tested in a treatment strategy to decrease damage and disease in lungs with pre-existing injury (Weiss et al., 2002; Huang et al., 2008; Piao et al., 2017).

Prostaglandins (PGs) are a group of lipid compounds that mediate inflammatory responses (Ricciotti and FitzGerald, 2011). They are converted from arachidonic acid through various PG synthases, the rate-limiting enzymes in the cyclooxygenase pathway of arachidonic acid metabolism (DeWitt, 1991). Multiple observations have indicated that PGs, such as PG E2 (PGE_2_) and PG D2 (PGD_2_), have anti-inflammatory and protective effects on ARDS (Birukova et al., 2007; Scher and Pillinger, 2009; Murata et al., 2013). Thus, gene transfer of PG synthase has been evaluated to upregulate endogenous PGs for treating lung injury. This approach worked in protection studies to reduce bleomycin-induced lung injury, but more importantly, retrovirally introduced PGD2 synthase into murine lungs that were pre-injured by bleomycin remarkedly increased animal survival rate and reduced edema accumulation, leukocyte infiltration, and plasma extravasation (Ando et al., 2003).

Another interesting approach has been to mine high-throughput sequencing analyses to identify potential therapeutic targets for ARDS. A recent genome-wide association study (GWAS) identified the non-receptor cytosolic tyrosine kinase FER as being associated with increased survival in patients with pneumonia and sepsis (Rautanen et al., 2015). In a mouse model of combined lung contusion and pneumonia, the expression of FER was down compared to controls. When FER was overexpressed in the lungs by electroporation-mediated gene transfer, survival of the animals was improved and antibacterial response genes were activated (Dolgachev et al., 2016). In follow-up studies, it was shown that in a Klebsiella pneumonia model in mice, electroporation-mediated delivery of FER plasmids to the lungs of mice previously infected with Klebsiella not only improved survival but also reduced bacterial counts in the lungs. Further experiments suggested that FER gene transfer activates the STAT pathway to enhance innate immunity and accelerate bacterial clearance in the lung (Dolgachev et al., 2018).

Another strategy to fight against the overwhelming inflammation in early ARDS is to suppress the proinflammatory effects by silencing genes that directly regulate the production of proinflammatory cytokines and chemokines or indirectly promote inflammation through RNAi (Lomas-Neira et al., 2008). Intratracheal delivery of siRNA could target the local lungs with high efficiency (Lomas-Neira et al., 2005). NF-κB is an important inflammation inducer that regulates the transcription of a number of downstream proinflammatory cytokines or mediators, such as IL-1b, IL-6, and TNF-α (Jha and Das, 2017). In recent studies, siRNA-mediated local or systematical silencing of NF-κB significantly protected animals against further increased proinflammatory cytokine (TNF-α, IL-6) release, histological and other lung injury scores in animals subsequently injured with LPS compared with injury and scramble control, indicating that NF-κB could be a potential target for developing RNAi gene therapy for ARDS (Jin L.-Y. et al., 2014; Li et al., 2016). A decoy strategy has also been employed to target the NF-κB inflammatory pathway, to block the binding of NF-κB to promoter regions of its targeted genes, resulting in the inhibition of proinflammatory gene transcription. In this approach, multiple copies of oligonucleotides encoding the NF-κB consensus binding site are delivered to cells or tissues to compete for activated NF-κB binding, thereby reducing normal signaling. In a sepsis model of cecal ligation and puncture (CLP), tail-vein injection of synthetic double stranded oligodeoxynucleotides (ODNs) to decoy NF-κB showed protective effects on septic lung injury, such as decreased transcription of sepsis-induced proinflammatory genes (iNOS, COX-2), decreased histological damage in terms of alveolar wall thickening, immune cell infiltration and hemorrhage, decreased vascular permeability, and improved blood gas exchange capacity (Matsuda et al., 2005). Other proteins that have a potential for siRNA therapeutics include neutrophil chemo-attractant Keratinocyte derived-chemokine (KC) and macrophage inflammatory protein-2 (MIP-2), which alleviate neutrophil activation-induced excessive inflammation and injury (Lomas-Neira et al., 2005). Similarly, in a hemorrhage induced sepsis mouse model, intratracheal delivery of siRNA to locally silence chemoattractant cytokines KC and MIP-2 significantly suppressed neutrophil influx into the lungs and decreased tissue or plasma level of IL-6, MIP-2 and MPO, activity (Lomas-Neira et al., 2005).

### Gene Delivery to Restore Alveolar Capillary Barrier Function

Alveolar capillary barrier disruption is a primary cause of ARDS leading to the influx of protein-rich fluid into alveoli and accumulation of pulmonary edema. Damage to junctional structures between epithelial cells and/or between endothelial cells, and cell death both result in alveolar capillary barrier dysfunction.

In recent years, gene delivery of target proteins to repair lung barrier function has been investigated. The Na^+^, K^+^-ATPase has been primarily studied for its function in promoting AFC, although increasing evidence demonstrates that the expression and function of the Na^+^, K^+^-ATPase are also closely involved in the regulation of epithelial barrier integrity (Rajasekaran et al., 2001b; Rajasekaran and Rajasekaran, 2003; Vadasz et al., 2007). For example, the Na^+^, K^+^-ATPase β1 subunit has been shown to be necessary for membrane localization of TJs, e.g., ZO-1, occluding. Furthermore, silencing of the β1subunit by siRNA disrupts the continuous staining pattern of ZO-1, indicating that the β1subunit might be directly or indirectly associated with TJ proteins (Madan et al., 2007; Rajasekaran et al., 2007). The Na^+^, K^+^-ATPase is required for establishing epithelial polarization and co-expression of the Na^+^, K^+^-ATPase β1 subunit, and E-cadherin has been shown to recover the lost polarity and junctions in MSV-MDCK cells, a highly invasive cell line (Rajasekaran et al., 2001b). In addition, the basolateral membrane-localized Na^+^, K^+^-ATPase acts as an adhesion molecule, forming a trans-dimer junction structure between cells. This structure appears dependent on the N-glycosylation of the β1 subunit’s extracellular domain (Vagin et al., 2012). Because of the dual role of Na^+^, K^+^-ATPase in enhancing both AFC and epithelial barrier function, this transporter has been targeted for gene delivery to treat ALI in various experimental animal models. Recent data from our laboratory showed that overexpression of the rat or human Na^+^, K^+^-ATPase β1 subunit upregulated the protein expression and membrane localization of TJ proteins occludin and ZO-1 (Lin et al., 2016; Bai et al., 2021). Furthermore, in cultured monolayers of rat alveolar epithelial ATI cells, overexpression of the β1 subunit increased transmembrane epithelial electrical resistance (TEER) by 30% compared to untransfected cells or those transfected with empty plasmids (Bai et al., 2021). More importantly, electroporation-mediated gene delivery of the rat or the human β1-subunit of the Na^+^, K^+^-ATPase to the lungs of mice with existing LPS-induced lung injury rescued experimental ALI by upregulating both AFC and TJ protein abundance, and pulmonary barrier function, as demonstrated by decreased lung permeability, total protein, and cellularity in BAL fluid, and improved overall histological injury outcomes (Mutlu et al., 2007; Lin et al., 2016).

Based on these findings, our laboratory pursued two avenues for further treatment: evaluation of the ability of gene transfer of individual tight junction protein genes to protect and treat ARDS in animal models, and identification of the pathway(s) by which the β1-subunit of the Na^+^, K^+^-ATPase upregulates barrier function. Occludin plays a central role in the formation, maintenance, regulation, and structure of tight junctions. In unpublished studies, when an occludin-expressing plasmid was delivered to the lungs of mice that had been injured 24 h previously with intratracheal LPS, occludin was overexpressed, which resulted in decreased pulmonary edema, improved histology, and reduced inflammation (numbers of infiltrating neutrophils and reduced levels of proinflammatory cytokines), compared to animals that received empty plasmids or no treatment at all (Lin et al., submitted for publication). However, it was not determined whether expression was localized to the alveolar epithelium or the capillary endothelium. Either way, these results suggest that gene therapy to increase levels of tight junction proteins directly may be a viable approach to treat ARDS.

Claudin family TJ proteins are critical components that are required to form apical junction complexes for alveolar epithelial barrier function. The major claudins expressed in ATI and ATII cells include claudins 3 and 4 (Frank, 2012). Although claudins 3 and 4 are highly homologous in a peptide sequence, they have opposite effects on the regulation of alveolar barrier function: overexpression of claudin 4 leads to decreased epithelial permeability and increased TEER, while opposite responses are observed for claudin 3 (Mitchell et al., 2011). In response to various injury-inducing stimuli (e.g., hyperoxia, ventilation, and septic shock), claudin 4 expression has been shown to be downregulated in mice, although an early upregulation of claudin 4 mRNA level was observed at 4 h in a ventilation-induced ALI mouse model (Wray et al., 2009; Cohen et al., 2010; Herrero et al., 2018; Vyas-Read et al., 2018). So far, there is no direct *in vivo* evidence indicating that overexpression of claudin 4 could benefit or protect from injury in living lungs, but claudin 4 could be a potential target for ARDS by gene therapy. Taken with the fact that levels of claudins show a positive correlation with AFC in human lungs, it suggests that this approach may have merit (Rokkam et al., 2011). In addition, claudin 5 is a primary tight junction component expressed in pulmonary endothelial cells (Kaarteenaho-Wiik and Soini, 2009) and has been shown to be downregulated in the lungs during influenza infection and other models of ALI (Armstrong et al., 2012). In several cell culture experiments, the overexpression of claudin 5 significantly protected endothelial cells from LPS-induced decreased permeability to molecules and restored TEER in these cells, indicating a role for claudin 5 in barrier protection and pulmonary leakage (Soma et al., 2004; Armstrong et al., 2012). The therapeutic potential of claudin 5 was further confirmed in a study investigating the beneficial effects of simvastatin on ALI, which concluded that claudin 5 is an important mediator of ALI protection by simvastatin (Chen et al., 2014). These studies indicate that claudin 5 might be a promising target for gene therapy for lung barrier protection.

Claudin-18 is another major tight junction protein expressed specifically in alveolar epithelial cells. So far, there has been no study directly reporting the effects, of claudin-18, by gene delivery, on ALI in an animal model. This is probably due to the complex role of claudin-18 in edema resolution. Claudin-18-knocked out mice did not exhibit apparent respiratory dysfunction and showed unchanged wet-to-dry weight ratios from baseline, although claudin-18 was downregulated at the protein and transcriptional levels during injury (Ohta et al., 2012; Li et al., 2014). However, the claudin-18-knocked out mice showed increased lung permeability to ions and solutes of various sizes, significantly enhanced fluid clearance rates with increased ion transport activities, and altered expression of claudins 3 and 4 (LaFemina et al., 2014; Li et al., 2014). These results suggest that the overexpression of claudin-18 may have actually exacerbated the injury, although because of its ability to regulate other claudins, cytoskeletal organization, b_2_ adrenergic signaling, and CFTR activity, it is difficult to say what ultimate effects its overexpression could have.

Fas/FasL apoptosis signaling has been identified as a main form of lung epithelial cell death, leading to alveolar capillary barrier damage. The Fas/FasL system is significantly upregulated in the BAL fluid of ARDS patients (Albertine et al., 2002). Using a FasL analog to compete with FasL for Fas binding improved pneumococcal bacterial clearance from the lungs of mice, indicating that the Fas/FasL axis might have a therapeutic potential (Matute-Bello et al., 2005b). In another study on hemorrhage-induced septic ALI, intratracheal instillation of siRNA 4 h after hemorrhagic shock and sepsis induction to silence local Fas in the lung showed markedly decreased levels of cytokines, such as TNFα, IL-6, and IL-10, and caspase 3 activity, indicating a protective effect by blocking Fas/FasL (Perl et al., 2005). Although this study primarily targeted local alveolar epithelial cells, the Fas receptor is also expressed in vascular endothelial cells, resulting in endothelial apoptosis (Hotchkiss et al., 2002). Thus, targeting Fas expression in the vascular endothelium for ARSD or sepsis endothelial injury might be a possible direction for future study.

Lung microvascular endothelial dysfunction plays an important role in the pathogenesis of ARDS and sepsis, and numerous approaches to modulate endothelial cell activation and decrease vascular leakage have been and are being investigated. For example, the bioactive sphingolipid metabolite sphingosine-1-phosphate (S1P) and its receptor, S1PR, have been reported to have protective effects on the endothelial barrier. S1P-mediated cellular events induce MLC phosphorylation, activation of Rho GTPase, and recruitment and assembly of adhesion junction molecules. Furthermore, genetic variants of the S1P receptor, such as S1PR3, have been shown to be positively associated with risk of ARDS (Natarajan et al., 2013). A recent publication reported that S1P receptor 1 (S1PR1) deletion resulted in increased endothelial permeability, and that S1PR1-expressing endothelial cells are required for barrier repair, suggesting a therapeutic potential of the S1P pathway in endothelial barrier dysfunction (Akhter et al., 2021).

Angiopoietin-1 (Ang-1) is an agonist for the endothelial-specific receptor tyrosine kinase Tie2 and has been shown to protect from microvascular endothelial permeability and plasma leakage both *in vitro* and *in vivo*. The Ang-1-Tie2 axis signals to downstream effector proteins and regulates intercellular junction complexes, like VE-cadherin or other adhesion molecules (e.g., PECAM-1, ICAM-1, VCAM-1, and E-selectin) (Gamble et al., 2000; Parikh, 2017). The anti-inflammatory role of Ang-1 has also been shown to be due to its suppression of neutrophils’ adherence to the endothelium during transmigration in ALI (Gamble et al., 2000). In mice with LPS-induced lung injury, Ang-1 expression was decreased in the lungs (Karmpaliotis et al., 2002). In mouse models of endotoxin-induced septic shock, animals pre-treated with adenoviral-mediated delivery of Ang-1 showed more resistance to subsequent injury, demonstrating significantly decreased lung edema, vascular permeability, histological damage, and mortality rate (Witzenbichler et al., 2005; Huang et al., 2008).

Mesenchymal stem cell (MSC)-mediated gene therapy has been reported to have a promising therapeutic potential for treating diseases such as sepsis. There are several advantages in using MSC therapy to treat ALI and sepsis. MSCs can be stably expanded *in vitro* to produce sufficient quantities for use while maintaining an undifferentiated state (Laffey and Matthay, 2017). This means that from another perspective, delivering target genes to manipulate endothelial damage, inflammation, or vascular injury could be more easily achieved through plasmid transfection of MSCs *in vitro*. In recent studies, systemic delivery of MSCs that overexpress an Ang-1 transgene to animals before LPS injury showed protective effects such as decreased lung leakage of plasma protein and decreased neutrophil infiltration (McCarter et al., 2007; Xu et al., 2008). Although these are promising results, there are fewer studies showing that Ang-1 overexpression could benefit the pre-injured lungs in animals. In addition, potential side effects of Ang-1 treatment were also reported (Sullivan et al., 2003; Long et al., 2008). Second, MSCs can easily access and traverse the vascular endothelium and incorporate into injured lungs *via* intravenous infusion, all while maintaining the ability to divide and self-renew. In addition, MSCs have good safety records with minimal immunogenicity, which helps avoid immune responses, a common adverse effect seen during viral vector-mediated gene delivery (Laffey and Matthay, 2017).

Several studies have reported that MSC-mediated gene therapy could repair alveolar capillary barrier disruption by targeting various genes. Ang-1 is the most studied one. Administration of MSCs overexpressing Ang-1 decreased IgM and albumin levels in BAL by more than 50% compared to LPS-induced lung hyperpermeability alone in mice, indicating significant restoration of lung barrier function (Mei et al., 2007). Growth factor genes, e.g., fibroblast growth factor (FGF), keratinocyte growth factor (KGF), and vascular endothelial growth factor (VEGF), also show a therapeutic potential for MSC-mediated gene therapy (Lee et al., 2011). FGF-activated signaling is required for the maintenance of interendothelial adhesion, the inhibition of which results in dissociation of the VE-cadherin/p120-catenin complex and disassembly of adherens and tight junctions, and eventually leads to endothelial barrier disruption and vascular leakage (Murakami et al., 2008). Moreover, FGF signaling is necessary for glycocalyx reconstitution, protecting against glycocalyx shedding during sepsis (Yang et al., 2017). Tail vein injection of MSCs overexpressing FGF2 into mice in an LPS-induced sepsis model significantly decreased the level of total protein and cytokines in BAL, lung edema, and histological lung damage, indicating the treatment potential of MSC-mediated FGF in lung injury (Zhao et al., 2015). KGF signaling is critical for pulmonary epithelial repair and proliferation. In animal lungs injured by intratracheal LPS administration, KGF gene-modified MSCs protected lung permeability, significantly decreased total protein levels in BAL, decreased edema (wet to dry ratio), attenuated inflammation with decreased expression of cytokines (IL-1β and TNF-α) and myeloperoxidase (MPO) activity in BAL fluid, and increased animal survival (Chen et al., 2013). Finally, VEGF is notable as a vascular permeability factor that has been shown to induce endothelial barrier disruption. In addition, paracrine factors secreted by MSCs, such as hepatocyte growth factor (HGF) and VEGF, were also investigated as having protective effects on endothelial barrier integrity in experimental LPS-induced lung injury models (Yang et al., 2015, 2016).

As a second approach to repair barrier function in ARDS, our lab has worked to identify the pathway(s) by which the β1 subunit of the Na^+^, K^+^-ATPase upregulates barrier function in cells and mouse models. Using a proteomics approach, we recently demonstrated that the novel CDC42-related kinase MRCKα is a specific interacting partner of the Na^+^, K^+^-ATPase β1-subunit, and that it is responsible for the upregulation of tight junction protein abundance in the membrane and activity seen following β1 overexpression (Figure 2; Bai et al., 2021). Interestingly, MRCKα expression appears down in the lungs of patients with ARDS compared to control lungs (Bai et al., 2021). In cultured rat ATI cells, we demonstrated that MRCKα was both necessary for β1’s ability to upregulate tight junction expression and TEER in cells using siRNA to knock down MRCKα expression and using specific inhibitors of the kinase (Bai et al., 2021). Furthermore, we also showed that the overexpression of MRCKα itself was sufficient to increase TEER seen in the monolayers. More recently, we have asked whether MRCKα overexpression in the lungs of mice in which lung injury had been previously induced by administration of LPS and have found that the overexpression of MRCKα leads to the same upregulation of tight junction proteins as does the transfer of the β1 subunit of the Na^+^, K^+^-ATPase, and provides the same degree of treatment of the disease, reducing pulmonary edema, lung permeability, measures of inflammation, and histological health of the lungs (Liu and Dean, 2021). As expected, the overexpression of MRCKα had no effect on the rates of AFC in mice. Taken together, these results suggest that repair of the epithelial and endothelial barrier activities is more important than upregulation of AFC alone.

While mouse models are often the first test of a given gene therapy, or for any therapeutic, approach for utility *in vivo*, they do not always reflect what will actually be of use therapeutically in patients. Indeed, this has been a major problem in developing effective treatments for ARDS in the past. An animal model that accurately duplicates the complex inflammatory and hemodynamic response that occurs in humans over several days during the development of sepsis-induced ARDS is critical to the understanding and treatment of lung injury. It has been shown that the success of clinical trials testing sepsis therapies depends on sound preclinical data using appropriate animal models (Piper et al., 1996). The best model, termed a “good evidence” model, is one in which the results of the study would be similar in a clinical trial (Piper et al., 1996). For example, while studies testing anti-endotoxin HA-1A demonstrated improved survival in a murine model of sepsis (Teng et al., 1985), a phase III clinical trial did not show survival benefit (Ziegler et al., 1991). However, when a clinically relevant “good evidence” large animal model of chronic septic shock was utilized to test the efficacy of HA-1A, the results were very similar to those of the clinical trial (Quezado et al., 1993). Similarly, a provocative article in PNAS several years ago generated great interest and press since it suggested that findings from mouse models alone do not always translate to larger species or humans (Seok et al., 2013).

There is a consensus that chronic, insidious-onset large animal models that mimic the pathogenesis of sepsis-induced ARDS are superior to small animal models as predictors of clinical efficacy (Parker and Watkins, 2001). To this end, Nieman et al. developed a chronic (48 h) two-hit sepsis and gut ischemia/reperfusion porcine model that accurately resembles the pathologic progression from injury to systemic inflammatory response syndrome, to septic shock, and finally to ARDS seen in human patients (Steinberg et al., 2005). Following injury, the animals are maintained and sedated according to the ARDSnet treatment paradigm (Network, 2000), making comparisons to existing human clinical trial data, more relevant and clear. Since sepsis is the leading cause of indirect ARDS, this model is highly germane developing any ARDS treatment (Sigurdsson et al., 2013). This model contains all of the key components of a “good evidence” animal model, such as a randomized, controlled study with supportive therapy (fluids, antibiotics) in a large animal, insidious-onset, chronic model of septic shock and ARDS (Piper et al., 1996). Moreover, the injury induced in this model yields all the features of ALI identified in the ATS consensus report on ALI in animals (Matute-Bello et al., 2011). The data generated from this model, which includes inflammatory mediator response, hemodynamic measures, lung function, and blood chemistry have allowed us to demonstrate that electroporation-mediated gene delivery of ENaC and Na^+^, K^+^-ATPase subunits 4 h after injury provides effective treatment of lung injury and statistically significant survival benefit in the pig.

**FIGURE 4:**
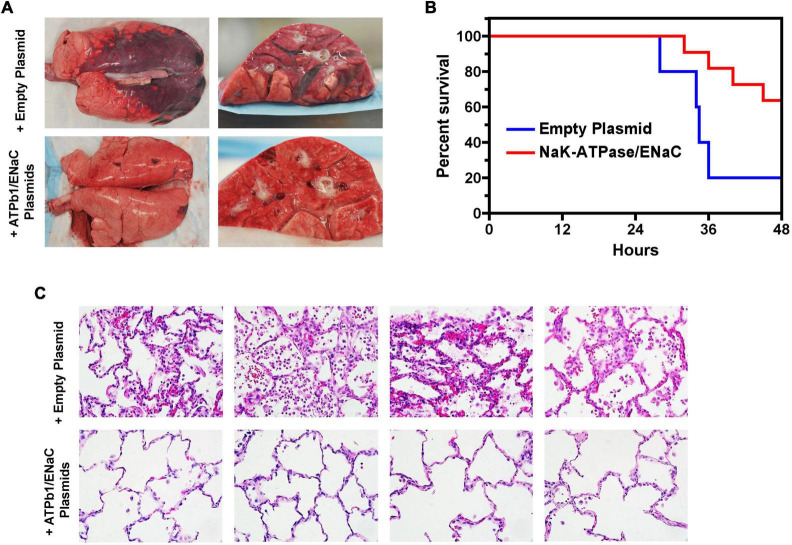
Electroporation-mediated gene transfer of Na^+^,K^+^-ATPase β1 subunit and α-ENaC plasmids can treat lung injury in a severe septic pig model of ARDS. Lung injury was induced in pigs (40 kg) at *t* = 0 by inducement of a fecal clot into the abdomen and ischemia-reperfusion injury of the superior mesenteric artery for 30 min and 4 h later, either empty plasmids or plasmids expressing α-ENaC and the Na^+^, K^+^-ATPase β1 subunit (ATP1b1) were delivered to the right and left lower lobes by electroporation. ARDSnet ventilation and vasopressor protocols (Network, 2000) were followed until death or the 48-h pre-set endpoint. **(A)** Gross histology of lungs from both groups of animals. Note the high degree of atelectasis and glossy presence of excess fluid in lungs from animals receiving empty plasmids. **(B)** Histology of lungs shows a much lower degree of injury in animals receiving the plasmid treatment. **(C)** Survival curves show that gene transfer of Na^+^, K^+^-ATPase β1 and α-ENaC plasmids increases survival (Dean, unpublished).

Emr et al. (2015) transferred a mixture of two plasmids expressing GFP-tagged rat Na^+^, K^+^-ATPase β1 subunit and DDK-tagged α1 subunit of ENaC, or a non-coding, empty plasmid as control, to pigs in this two-hit model of lung injury (Figure 4). Four h after injury (to mimic when a patient would present in the ER after traumatic injury leading to ALI), a bronchoscope was used to deliver 50 ml of DNA in saline (50 mg endotoxin-free plasmid), each into the left lower and right lobes, within a 2-min period. The bronchoscope was removed, and the animals were electroporated using external defibrillator pads placed on either side of the chest (“external electroporation”). Alternatively, in several experiments, the bronchoscope was left in the lung and used as an internal electrode coupled to one external electrode (“Internal electroporation”). Eight pulses (2,000 V at 150 μs each; ∼150 V/cm) were then applied to the animals. The anesthetized animals were maintained on mechanical ventilation and administered vasopressors according to the human ARDSnet and Early Goal Directed Therapy protocols (Network, 2000; Rivers et al., 2001). Initially, the animals were ventilated at 10 cc/kg tidal volume (V_T_) with a PEEP of 5 cmH_2_O, respiratory rate of 12, FiO_2_ 30%, and inspiratory to expiratory Ratio 1:2. The animals were transitioned to low V_T_ (6 cc/kg) when they met ARDSnet clinical criteria of PaO_2_/FiO_2_ < 300 (mild ARDS) the per ARDSnet protocol (Network, 2000). Appropriate adjustments were made to maintain adequate minute volume. PEEP and FiO_2_ were adjusted in response to changes in SaO_2_ along the “High PEEP, Low FiO_2_” scale (Network, 2000). If airway plateau pressure (P_plat_) rose above 30 cm H_2_O, V_T_ was further reduced by 1 cc/kg increments to 4 cc/kg with appropriate adjustments in respiratory rate to maintain equivalent minute volume per ARDSnet guidelines. The upper limit for respiratory rate was 35 BPM, with titrations made in V_T_ if respiratory acidosis was detected (pH < 7.15) according to the ARDSnet protocol. Broad-spectrum antibiotics (ampicillin 2 g IV and metronidazole 500 mg IV) were given following abdominal closure and every 12 h until the end of the study. The results seen following electroporation-mediated gene transfer of the Na^+^, K^+^-ATPase and ENaC genes were remarkable (Figure 4).

First, gene transfer of an empty plasmid (pCDNA3) did not change the course of the injury compared to animals with no intervention. In contrast, the transfer of Na^+^, K^+^-ATPase/α-ENaC plasmids lead to improved lung function, improved kidney function, less injured lungs upon gross and microscopic histological analysis, and greater survival. In terms of lung function, electroporation of the treatment plasmids increased P/F ratios and compliance in the pigs, while the amount of PEEP needed to keep the lungs open was less in these animals, as was plateau pressure. As in mice, gene transfer of Na^+^,K^+^- ATPase/α-ENaC plasmids increased the abundance of tight junction proteins ZO-1 and occludin in the septic pigs (Emr et al., 2015; Lin et al., 2016). This upregulation may indeed result in improved barrier function and may account in part for the improved outcome. Gene transfer also had an effect on cellular death with 3.5 ± 1.4% (mean ± SD) of alveolar cells staining tunnel positive for apoptosis in empty plasmid-treated pigs compared to 1.9 ± 0.9% in Na^+^,K^+^-ATPase/α-ENaC pigs (*p* < 0.01). The health of the lungs was evident upon visual inspection of lungs removed from the euthanized animals (Emr et al., 2015). Histologically, the animals receiving either no intervention or empty plasmid showed all the signs of ALI/ARDS, namely, thickened alveolar septa, cell infiltrates, hemorrhage, edema, fibrin deposits, hyaline membranes, and vascular congestion, while animals receiving the Na^+^,K^+^-ATPase/α-ENaC plasmids presented much more like healthy lungs (Figure 4). Quantitative histological analysis revealed statistically significant differences between these groups (Emr et al., 2015). Wet to dry ratios showed significantly drier lungs in the animals receiving the Na^+^,K^+^-ATPase/α-ENaC plasmids. Finally, survival was also improved. The success of this electroporation-mediated gene therapy in such a stringent model demonstrates the robust nature and promise of this approach for possible use in critically ill patients.

## Conclusion

Despite the fact that there has been no single gene or combination of genes identified that are responsible for ARDS, a number of genes have been effectively targeted for up- or down-regulation in multiple animal models that have shown varied degrees of alleviation of many of the symptoms and severity of ARDS. While in the past, most focus has been aimed at increasing the expression of ion channels and transporters to aid in alveolar fluid removal, these have not been as successful in treatment studies. More promising strategies are targeted toward dampening inflammatory responses and repairing or strengthening the alveolar-capillary barrier. In both cases, a number of genes have shown promise.

Perhaps the major problem with developing treatments for ARDS at the gene therapy level has been that most studies have relied on prevention approaches as opposed to treatment. In these instances, genes are transferred to healthy lungs before any lung injury is induced. While this allows for maximal gene delivery, since healthy lungs allow for greater distribution of any viral or nonviral gene delivery agent and maximal transcription from delivered transgenes, it is not reflective of how any gene therapy or other treatment would be given clinically (i.e., after development of lung injury). Furthermore, a myriad of studies have clearly shown that mice are not humans, and that ALI and ARDS in mice do not show all of the same pathological hallmarks as the human disease. Larger animals that more closely reflect human pathophysiology are needed to make any advancement toward the clinic. Thus, greater emphasis should be placed on those studies that (1) use experimental designs aimed at treating existing disease and (2) use larger, clinically relevant animal models of ARDS.

Using a number of different animal models and humans, many of the molecular mechanisms that contribute directly and indirectly to the pathogenesis of ARDS have been identified over the past 30 years. Developing strategies to treat this and any disease at the genetic level using gene therapy is perhaps the most direct way to change the pathophysiology and resolve injury, if concerns for safety and appropriate gene expression are kept in balance. While a number of different families or types of targets have been studied (inflammatory, edema clearance, and barrier, etc.), perhaps the major limitation for effective gene therapy remains optimizing gene delivery itself. Gene delivery systems that are nontoxic and non-inflammatory allow for repeat dosing and produce therapeutic levels of gene product, all the while being targeted to desired cell types is needed. Although each different viral vector or nonviral chemical or physical technology may address one or more of these problems, none has yet solved all of them. However, advances are rapid in both the areas of gene delivery and our understanding of the disease itself, so it is highly likely that safe and efficacious treatments for ARDS are within reach.

## Author Contributions

JL and DD contributed to, wrote, revised the manuscript, and approved the submitted version.

## Conflict of Interest

DD has applied for patent protection for the use of MRCKa in the gene therapy treatment of ARDS. DD was also a consultant for several non-viral gene therapy companies providing guidance in the area of vector design, but not for any aspect of using gene therapy to treat lung diseases, including ARDS. The remaining author declares that the research was conducted in the absence of any commercial or financial relationships that could be construed as a potential conflict of interest.

## Publisher’s Note

All claims expressed in this article are solely those of the authors and do not necessarily represent those of their affiliated organizations, or those of the publisher, the editors and the reviewers. Any product that may be evaluated in this article, or claim that may be made by its manufacturer, is not guaranteed or endorsed by the publisher.
